# Solvent-Free Synthesis of Chiral Substituted *N*‑Benzylideneanilines Imines: X‑ray Structure, and DFT Study

**DOI:** 10.1021/acsomega.5c06111

**Published:** 2025-10-22

**Authors:** Guadalupe Hernández Téllez, José A. Reyes-Avendaño, José M. Bravo-Arredondo, Gloria E. Moreno Morales, Pankaj Sharma, Claudia P. Villamizar C., Angel Mendoza, Bertin Anzaldo

**Affiliations:** † 3972 Benemérita Universidad Autónoma de Puebla, Laboratorio de Síntesis de Complejos, Facultad de Ciencias Químicas, Edificio FCQ-6, Ciudad Universitaria, Av. San Claudio y Blvd. 14 Sur, Col. San Manuel, C.P. 72592, Puebla, Pue., México; ‡ Benemérita Universidad Autónoma de Puebla, Facultad de Ingeniería Química, Apartado Postal J-48, C. P. 72570 Puebla, Puebla, México; § Benemérita Universidad Autónoma de Puebla, Posgrado en Dispositivos Semiconductores, Prolongación 14 Sur, IC5, Puebla, Puebla 72570, Mexico; ∥ Universidad Autónoma de Tlaxcala, Facultad de Ciencias Básicas, Ingeniería y Tecnología, C.P. 90401 Apizaco, Tlax., México; ⊥ Instituto de Química-UNAM, Circuito exterior, C.U. Coyoacán, C.P. 04510 Ciudad de México, México; # Centro de Química del Instituto de Ciencias, Benemérita Universidad Autónoma de Puebla, 18 Sur y Av. San Claudio, Col. San Manuel, Puebla 72570, México

## Abstract

Solvent-free synthesis of five chiral-substituted *N*-benzylideneanilines (**I–V**) was reported in high yields, and all these imines were characterized by various physicochemical analysis techniques. Crystallographic analysis revealed that variations in crystal packing were primarily influenced by steric effects rather than halogen substituents. The contribution of halogen bonding to the chromic properties of these compounds was systematically evaluated. Their electronic and optical properties were also investigated theoretically using density functional theory (DFT) calculations at the B3LYP and M06–2X levels of theory with the 6–311+G­(d,p) basis set, which reproduced the experimental structures with good accuracy. Using frontier molecular orbital analysis, two regimes were identified: imines **I**, **II**, and **IV** have localized HOMOs on the imine moiety that lead to larger HOMO–LUMO gaps and higher chemical hardness, while imines **III** and **V** have orbitals that are delocalized over extended π-systems with smaller gaps, lower softness, and higher polarizability. Trends were corroborated by global reactivity parameters which indicated that the charge-transfer ability of **III** and **V** is enhanced and TD-DFT calculations reproduced the experimental ultraviolet–visible (UV–vis) with red-shifted absorptions. ECD spectra showed stronger Cotton effects in **III** and **V** being attributed to their electronic delocalization and lower hardness. NBO analysis revealed stabilization via π­(C–C) → π*­(N–C) and n­(O/Cl) → π*­(C–C) interactions; NCI plots highlighted dispersive and C–H···π contacts. Complementary QTAIM analysis identified two weak Cl···Cl interactions in imine **IV**, indicating the effect of halogen contacts on supramolecular packing. This integrated experimental–theoretical study showed that substituents regulate electronic structure, charge-transfer capacity, and chiroptical activity, guiding future designs of imine-based optoelectronic and functional chiral materials.

## Graphical abstract

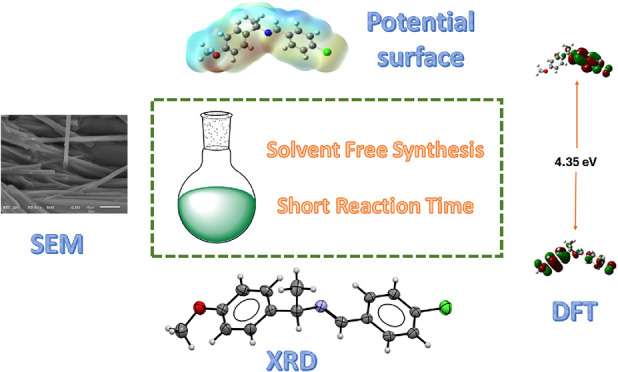

## 1 Introduction

Schiff bases are fundamental building blocks in organic chemistry, known for being versatile N-substituted imines with a [C=N] functional group, and are important in coordination chemistry, pharmaceuticals, and materials science.^1–4^ These imines are typically synthesized through a reversible condensation reaction between carbonyl compounds and primary amines where the electrophilic carbonyl carbon reacts with nucleophilic amines to form imine bonds (C=N). Recent advances in green chemistry have highlighted this methodology as an efficient and sustainable approach, emphasizing minimal environmental impact.^5–7^

In coordination chemistry, Schiff bases are particularly noteworthy due to the nucleophilic nitrogen atom of the imine group, which facilitates interactions with diverse metal centers and makes them important in different fields such as catalysis, optoelectronics, and the development of biologically active compounds.^8–11^ By carefully selecting suitable precursors and incorporating donor atoms (e.g., O, S, N, P), one can fine-tune the coordination environment of Schiff bases, thereby stabilizing metal centers across different oxidation states and geometries.^12–21^ Furthermore, the presence of electron-donating or withdrawing substituents allows fine-tuning of donor–acceptor interactions and π-conjugation, thereby enhancing their structural and electronic properties.^22–25^

When integrated into conjugated systems, they demonstrate tunable photophysical properties, while substituent electronic characteristics significantly influence stability.^26–29^ However, there has been limited investigation into how structural modifications, such as halogen substitution, affect the optical and electronic properties of Schiff base complexes.^30–34^

In this work, we describe the synthesis of chlorinated chiral imines with different substituents and their physicochemical characterization by various techniques. Experimental results indicate that halogen substitution in these chiral imines has an impact on spectroscopic and structural changes. Density Functional Theory (DFT) calculations were performed to complement the experimental findings, thereby providing a deeper understanding of electronic features of these chiral imines concerning HOMO–LUMO gap, molecular electrostatic potential (MEP) surfaces, global reactivity parameters (GRPs) like hardness, softness, electronegativity, electrophilicity, and how structural modifications affect the stability, charge-transfer capacity, and optical behavior. The theoretical geometries were confirmed by single-crystal X-ray diffraction structures, as well as the simulated infrared spectra. TD-DFT calculations were done to calculate ultraviolet–visible (UV–vis) spectra and compare them with experimental data. In addition, Natural Bond Orbital (NBO), Noncovalent Interaction (NCI), and Quantum Theory of Atoms in Molecules (QTAIM) analyses were also used to explain donor–acceptor interactions, intramolecular charge delocalization, and weak intermolecular forces that facilitate supramolecular stabilization.

## 2 Materials and Methods

### 2.1 Instrumentation

^1^H NMR and ^13^C NMR spectra were recorded on a Bruker-500 spectrometer (500 MHz). Chemical shifts are reported in ppm downfield relative to tetramethylsilane (TMS) as an internal standard (δ = 0.00). Chemical shift values are reported in parts per million δ­(ppm) and (*J*) values are in Hertz. The splitting pattern is indicated as abbreviations: singlet (s), doublet (d), doublet of doublet (dd), triplet (t), quartet (q), and multiplet (m). Mass spectra were obtained using the Electron Impact (EI) technique on a MStation JMS-700 spectrometer (JEOL Ltd., Tokyo, Japan) operated in positive-ion mode at 70 eV. Data are expressed in mass-to-charge (*m*/*z*) units. IR spectra were recorded on a PerkinElmer Spectrum One FT-IR spectrometer with a Universal ATR accessory (PerkinElmer, Waltham, MA). Optical rotation was measured using a PerkinElmer 341 polarimeter (PerkinElmer, Waltham, MA). Melting points were determined using an Electrothermal MEL-TEMP 3.0 apparatus (Electrothermal, Staffordshire, U.K.) and are uncorrected. All reagents were obtained from commercial suppliers and used without further purification. Solvents were purified by standard methods and freshly distilled before use. The absorbance spectra were obtained using a UV–Vis-NIR spectrophotometer from Cary 5000 Agilent Technologies. UV–vis-NIR Spectrophotometer running in double beam mode with a matched pair of quartz absorbance cuvettes (1 cm × 1 cm). For scanning electron microscopy (SEM) micrographs, a JEOL model microscope was used JSM-6610LV, with Tungsten filament. Samples were analyzed in a high vacuum and at a potential of 20 kV. The samples were mounted on carbon tape and coated with gold in a scan time of 60 s and ×200 and ×2000 amplification.

### 2.2 X-ray Crystal Structure Determination

Single-crystal X-ray diffraction data were collected using an Xcalibur Atlas Gemini Diffractometer. Mo Kα radiation was used for imines **I–III**, **V**, and Cu Kα radiation was used for imine **IV**. Data were measured at 298 K. Crystals were recrystallized by slow evaporation using CH_2_Cl_2_. The structure determination and refinement were performed using the SHELXL^35^ software suite and Olex2.^36^ Detailed refinement parameters and structural features are summarized in Table 1.

**Table 1:** Crystal Data and Refinement Parameters for Chiral Imines **I–V**

Imine	I	II	III	IV	V
CCDC Number	2291440	2417847	2291441	2299736	2417849
chemical formula	C_15_H_14_ClN	C_16_H_16_ClN	C_19_H_16_ClN	C_17_H_16_ClN	C_16_H_16_ClNO
molar mass (g mol^–1^)	243.72	257.75	293.78	269.76	273.75
*T* (K)	293(2)	293(2)	293(2)	293(2)	293(2)
crystal system	monoclinic	monoclinic	monoclinic	orthorhombic	orthorhombic
space group	*P*2_1_	P2_1_	*P*2_1_	*P*2_1_2_1_2_1_	*P*2_1_2_1_2_1_
*a* (Å)	6.5566(3)	6.8535(9)	8.2250(4)	5.14092(7)	6.5676(8)
*b* (Å)	7.6589(4)	7.5423(7)	7.3755(3)	11.30751(15)	7.5762(7)
*c* (Å)	13.5381(7)	14.1714(18)	13.4347(5)	24.5905(4)	29.183(2)
α (°)	90	90	90	90	90
β (°)	95.447(4)	97.439(12)	101.378(4)	90	90
γ (°)	90	90	90	90	90
*V* (Å^3^)	676.77(6)	726.37(15)	798.98(6)	1429.47(3)	1452.1(2)
*Z*	2	2	2	4	4
*p*_calc_ (g cm^–3^)	1.196	1.178	1.221	1.253	1.252
μ (mm^–1^)	0.260	0.245	0.232	2.225	0.255
*F*(000)	256.0	272.0	308.0	568.0	576.0
crystal size (mm^3^)	0.547 × 0.337 × 0.132	0.282 × 0.17 × 0.1	0.573 × 0.475 × 0.257	0.506 × 0.398 × 0.199	0.464 × 0.345 × 0.257
radiation (λ/Å)	Mo Kα (λ = 0.71073)	Mo Kα (λ = 0.71073)	Mo Kα (λ = 0.71073)	Cu Kα (λ = 1.54184)	Mo Kα (λ = 0.71073)
2θ range (°)	6.046–52.744	5.798–54.198	6.186–61.012	7.19–154.178	6.06–52.742
reflections collected	44501	5269	18159	15654	8935
*R*_int_	0.0729	0.0505	0.0309	0.0208	0.0325
GOF on *F* ^2^	1.048	0.961	1.022	1.043	1.101
*R*_1_, *w*R**_2_ (*I* ≥ *2*σ (I))	0.0641, 0.1688	0.0656, 0.1342	0.0487, 0.1047	0.0368, 0.1050	0.0522, 0.1119
*R*_1_, *w*R**_2_ (all data)	0.0847, 0.1955	0.1680, 0.1854	0.0859, 0.1241	0.0381, 0.1073	0.0794, 0.1235

### 2.3 Computational Methods

The molecular geometries of the chiral imines (**I–V**) were optimized from single-crystal X-ray diffraction structures using Density Functional Theory (DFT). All calculations were performed with the Gaussian 09 software package.^37^ The ground-state geometries and electronic properties were calculated using two exchange-correlation functionals: the B3LYP hybrid functional and the M06–2X meta-hybrid functional,^38^ both in conjunction with the 6–311+G­(d,p) basis set^39–41^ due to its balanced treatment of valence polarization and diffuse functions, making it particularly suitable for systems bearing electronegative atoms (such as nitrogen and chlorine), lone pair orbitals, and delocalized π-electrons, useful for our conjugated and chlorinated imines, and non metal-atoms.^42,43^ For all geometry optimizations, subsequent vibrational frequency calculations were accomplished to confirm the true minimum nature of the optimized structures with no imaginary frequencies and allowed for the prediction of IR spectra. Using the optimized geometries, Time-Dependent DFT (TD-DFT) calculations, at the same levels of theory, were carried out to determine optoelectronic properties, specifically UV–Vis absorption and electronic circular dichroism (ECD) spectra were simulated for each imine. Solvent effects were included via the polarizable continuum model (PCM), with acetonitrile (dielectric constant ε = 37.5) as the solvent. Additionally, the energy band gap (HOMO–LUMO), MEP surfaces, and GRPs such as hardness, softness, chemical potential, electronegativity, electrophilicity index, and polarizability were determined using DFT. These descriptors were chosen to systematically evaluate the substituent effects on the imine framework, providing a quantitative understanding of how electronic delocalization and donor–acceptor interactions influence stability and reactivity. The HOMO–LUMO gap offers direct insight into molecular stability and electronic transitions; the MEP surfaces map electrostatic potentials and predict sites of electrophilic and nucleophilic attack; while GRPs quantify overall reactivity trends and chemical softness/hardness. For the visualization of the optimized geometries, the molecular orbitals, and MEP surfaces the GaussView 5 ^44^ software was used. Natural Bond Orbital (NBO), noncovalent interaction (NCI), and QTAIM analyses were performed for all imines using Multiwfn 3.8 ^45^ and visualized with Visual Molecular Dynamics (VMD).^46^ Also, reduced density gradient (RDG) approach were done to visualize weak intra- and intermolecular forces in real space.

### 2.4 Synthesis

#### 2.4.1 General Methodology

Chiral imines (**I–V**) were synthesized via mechanochemical methods under solvent-free conditions at ambient temperature by the condensation reaction between 4-chlorobenzaldehyde (1.5 mmol) and equimolar amounts of primary amines: (*S*)-(−)-1-phenylethylamine, (*S*)-(−)-1-(4-methylphenyl)­ethylamine, (*S*)-(−)-1-(1-naphthyl)­ethylamine, (*S*)-(+)-1,2,3,4-tetrahydro-1-naphthylamine, (*S*)-(−)-1-(4-methoxylphenyl)­ethylamine, as illustration in Scheme 1. The synthesis was conducted at room temperature and solvent-free, facilitating high efficiency as well as easy separation and purification. The crude products obtained were recrystallized from CH_2_Cl_2_, affording the corresponding pure Schiff bases (**I–V**) with suitable crystals for the determination of single crystal structure.

**Scheme 1:**
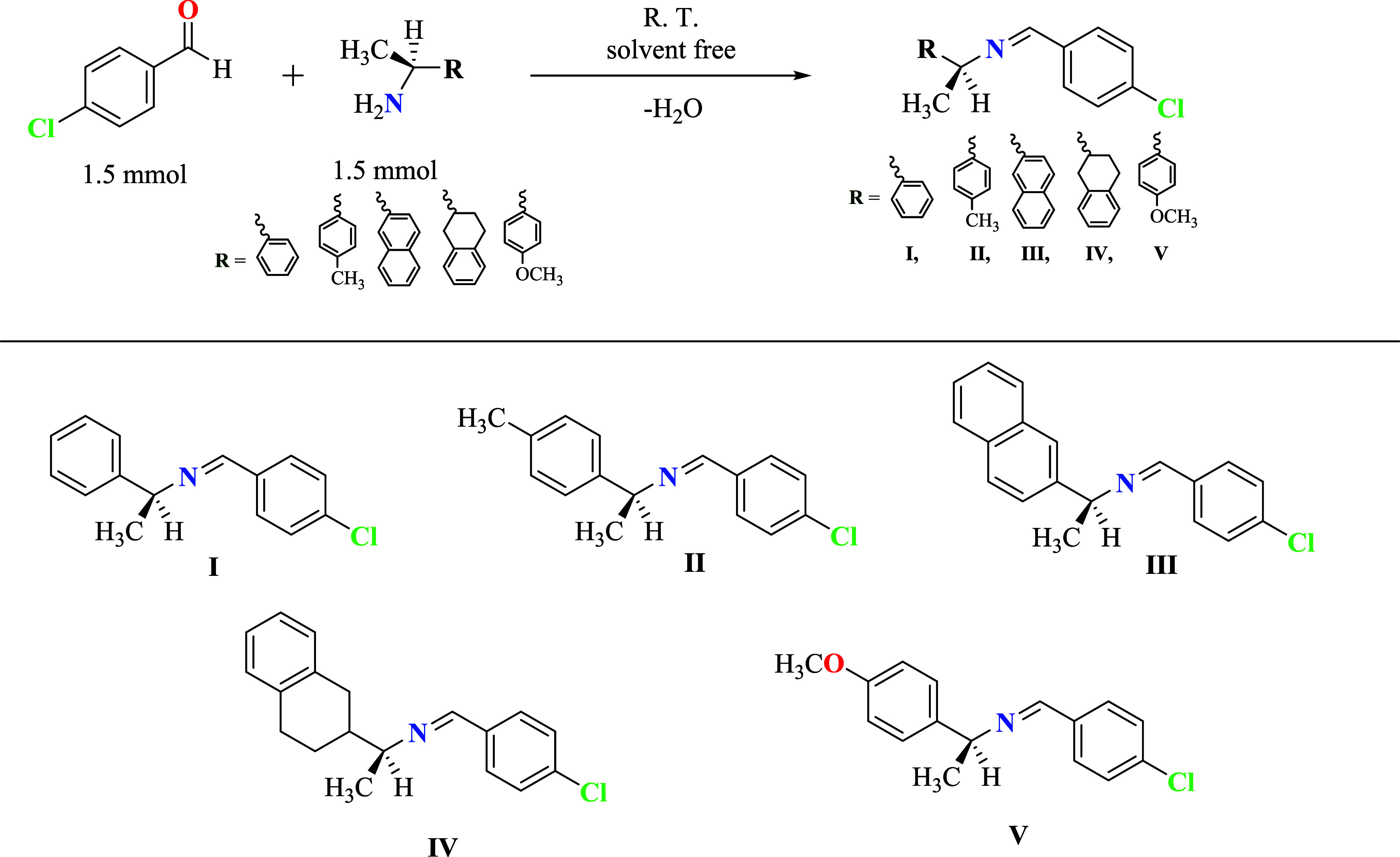
General Procedure for the Synthesis of Compounds **I–V**

**Imine I**: 4-chlorobenzaldehyde 0.210 g (1.5 mmol) and equimolar amounts of primary (*S*)-(−)-1-phenylethylamine 0.182 g (1.5 mmol), was added under solvent-free conditions. The imine was obtained as a light-yellow crystal. Yield: 96%. **Mp** 68–70 °C. **FT-IR** (ν, cm^–1^): 1638 (C=N). ^**1**^ **H NMR** (500 MHz, CDCl_3_/TMS, δ in ppm, *J* in Hz) = 8.32 (*s*, 1H; H-7, HC=N), 7.71 (*d*, *J*_*H–H*_ = 8.5, 2H, H-2, H-6), 7.44 – 7.30 (*m*, 6H; H-3, H-5, H-12, H-13, H-15, H-16), 7.24 (*t*, *J*_*H–H*_ = 6.7, 1.4, 1H, H-14), 4.53 (*q*, *J*_*H–H*_ = 6.7, 1H; H-9), 1.58 (*d*, *J*_*H–H*_ = 7.5, 3H; H-11). ^**13**^ **C** {^1^H} **NMR**(125 MHz, CDCl_3_/TMS, δ in ppm): 158.12 (HC=N, C-7), 144.98 (C-10), 136.51 (C-1), 134.88 (C-4), 129.48 (C-3, C-5), 128.83 (C-2, C-6), 128.50 (C-13- C-15), 126.97 (C-14), 126.64 (C-12, C-16), 69.80 (C-9), 24.88 (C-11). **MS–EI** ^**+**^ (*m*/*z*): calcd for C_15_H_14_ClN: 243.7340; found: 243. [α]_*D*_^20^= +97.3° (c = 1, CHCl_3_).

**Imine II**: 4-chlorobenzaldehyde 0.210 g (1.5 mmol) and equimolar amounts of primary (*S*)-(−)-1-(4-methyl)­phenylethylamine 0.203 g (1.5 mmol), was added under solvent-free conditions. The imine was obtained as a light-yellow crystals. Yield: 94%. **Mp** 74–76 °C. **FT-IR** (ν, cm^–1^): 1631 (C=N). ^**1**^ **H NMR** (500 MHz, CDCl_3_/TMS; δ: ppm, *J*:Hz): 8.30 (*s*, 1H; H-7, HC=N), 7.73–7.67 (*m*, 2H; H-3, H-5), 7.36 (*d*, *J*_*H–H*_ = 8.5, 2H; H-13, H-15), 7.30 (*d*, *J*_*H–H*_ = 8.1, 2H; H-2, H-6), 7.15 (*d*, *J*_*H–H*_ = 7.6, 2H; H-12, H-16), 4.51 (*q*, *J*_*H–H*_ = 6.6, 1H; H-9), 2.33 (*s*, 3H; H-18), 1.58 (*d*, *J*_*H–H*_ = 6.7, 3H; H-11). ^**13**^ **C**{^1^H} **NMR** (125 MHz, CDCl_3_/TMS, δ in ppm): δ 157.96 (HC=N, C-7), 141.95 (C-10), 136.56 (C-14), 136.44 (C-1), 134.92 (C-4), 129.47 (C-3, C-5), 129.18 (C-13, C-15), 128.80 (C-2, C-6), 126.55 (C-12, C-16), 69.54 (C-9), 24.76, (C-11). 21.10 (C-10); **MS–EI** ^**+**^ (*m*/*z*): calcd for C_16_H_16_ClN: 257.7610; found: 257. [α]_*D*_^20^ = +91.1° (c = 1, CHCl_3_).

**Imine III**: 4-chlorobenzaldehyde 0.210 g (1.5 mmol) and equimolar amounts of primary (*S*)-(−)-1-(1-naphthyl)­ethylamine 0.257 g (1.5 mmol), were added under solvent-free conditions. The imine was obtained as light-yellow crystals. Yield: 92%. **Mp** 94–96 °C. **FT-IR** (ν, cm^–1^): 1646 (C=N). ^**1**^ **H NMR** (500 MHz, CDCl_3_/TMS; δ: ppm, *J*:Hz): 8.37 (*s*, 1H; HC=N, H-7), 8.23 (*d*, *J*_*H–H*_ = 8.5, 1H; H-18), 7.87 (*dd*, *J*_*H–H*_ = 8.1, 1.6, 1H; H-14), 7.77 (*dd*, *J*_*H–H*_ = 8.5, 2H; H-13, H-20), 7.73 (*d*, *J*_*H–H*_ = 8.5, 2H; H-3, H-5), 7.54 (*ddd*, *J*_*H–H*_ = 16.7, 6.8, 1.5, 1H; H-19), 7.53–7.44 (*m*, 2H; H-12, H-21), 7.40–7.35 (*m*, 2H; H-2, H-6), 5.35 (*q*, *J*_*H–H*_ = 6.6, 1H; H-9), 1.73 (*d*, *J*_*H–H*_ = 6.7, 3H; H-11). ^**13**^ **C**{^1^H} **NMR** (125 MHz, CDCl_3_/TMS, δ in ppm): 158.34 (C-7), 140.92 (C-10), 136.55 (C-1), 134.96 (C-15), 134.01 (C-4), 130.62 (C-16), 129.50 (C-2, C-6), 129.00 (C-20), 128.84 (C-3, C-5), 127.47 (C-14), 125.90 (C-19), 125.70 (C-13), 125.40 (C-12), 124.03 (C-18), 123.56 (C-21), 65.59 (C-9), 24.50 (C-11); MS–EI^+^ (*m*/*z*): calcd for C_19_H_16_ClN: 293.7940; found: 293. [α]_*D*_^20^ = +257.7° (c = 1, CHCl_3_).

**Imine IV**: 4-chlorobenzaldehyde 0.210 g (1.5 mmol) and equimolar amounts of primary (*S*)-(+)-1,2,3,4-tetrahydro-1-naphthylamine 0.220 g (1.5 mmol) were added under solvent-free conditions. The imine was obtained as light-yellow crystals. Yield: 83%. **Mp** 52–53 °C. **FT-IR** (ν, cm^–1^): 1640 (C=N). ^**1**^ **H NMR** (500 MHz, CDCl_3_/TMS; δ: ppm, *J*:Hz): 8.36 (*s*, 1H; H-7, HC=N), 7.74–7.69 (*m*, 2H; H-3, H-5), 7.41–7.35 (*m*, 2H; H-2, H-6), 7.19–7.08 (*m*, 3H; H-18, H-19, H-20), 6.99 (*d*, *J*_*H–H*_ = 8.2, 1H; H-21), 4.52 (*t*, *J*_*H–H*_ = 6.4, 1H; H-9), 2.97–2.79 (*m*, 1H; H-14), 2.14–2.08 (*m*, 1H; H-12), 2.09–1.96 (*m*, 2H; H-13), 1.91–1.79 (*m*, 1H; H-12′). ^**13**^ **C**{^1^H} **NMR** (125 MHz, CDCl_3_/TMS, δ in ppm): 159.15 (C-7), 137.16 (C-15), 136.97 (C-16), 136.56 (C-1), 134.81 (C-4), 129.57 (C-3, C-5), 129.26 (C-21), 128.85 (C-2, C-6), 128.60 (C-18), 127.02 (C-19), 125.85 (C-20), 68.52 (C-9), 31.52 (C-14), 29.51 (C-12), 20.11 (C-13). **MS–EI** ^**+**^ (*m*/*z*): calcd for C_17_H_16_ClN: 269.7720; found: 269. [α]_*D*_^20^= – 16.5° (c = 1, CHCl_3_).

**Imine V**: 4-chlorobenzaldehyde 0.210 g (1.5 mmol) and equimolar amounts of primary (*S*)-(−)-1-(4-methoxylphenyl)­ethylamine 0.227 g (1.5 mmol), was added under solvent-free conditions. The imine was obtained as a light-yellow crystals. Yield: 90%. **Mp** 97–99 °C. **FT-IR** (ν, cm^–1^): 1641 (C=N). ^**1**^ **H NMR** (500 MHz, CDCl_3_/TMS; δ: ppm, *J*:Hz): 8.33 (s, 1H; H-7), 7.75–7.71 (*m*, 2H; H-3, H-5), 7.44–7.38 (*m*, 4H; H-2, H-6), 7.37–7.34 (*m*, 2H; H-12, H-16), 6.94–6.89 (*m*, 2H; H-13, H-15), 4.53 (*q*, *J*_*H–H*_ = 6.6, 1H; H-9), 3.82 (*s*, 3H; H-19), 1.59 (*d*, *J*_*H–H*_ = 6.6, 3H; H-11). ^**13**^ **C**{^1^H} **NMR** (125 MHz, CDCl_3_/TMS, δ in ppm): 158.55 (C-14), 157.86 (C-7), 137.05 (C-10), 136.44 (C-1), 134.90 (C-4), 129.46 (C-2, C-6), 128.80 (C-3, C-5), 127.78 (C-12, C-16), 113.85 (C-13, C-15), 69.14 (C-9), 55.30 (H-19), 24.72 (C-9). **MS–ESI** ^**+**^ (*m*/*z*): calcd for C_16_H_16_ClNO: 273.7600; found: 273. [α]_*D*_^20^ **=** + 69.8° (c = 1, CHCl_3_).

## 3 Results and Discussion

The imines (**I–V**) were synthesized by the reaction of 4-chlorobenzaldehyde with enantiopure chiral amines and were characterized by mass spectrometry, Fourier-transform infrared (FT-IR), ^1^H and ^13^C NMR spectroscopy. The mass spectra confirmed the presence of the molecular ion (M^+^), along with two principal fragmentation ions: [M–CH_3_]^+^ and [M– C_7_H_5_ClN]^+^ in all the imines.

In the FT-IR spectra, of all the compounds (**I–V**), strong absorption bands in the 1631–1647 cm^–1^ range were observed, which corresponds to the ν­(C=N) stretching vibration. In compounds **III** and **V**, this vibration appears at 1647 and 1641 cm^–1^, respectively, which is slightly at a higher frequency than in the other compounds (∼1638 cm^–1^). This shift is attributed to the electron-donating effects of the methoxy (−OCH_3_) group (compound **III**) and the naphthalene moiety (compound **V**). Additionally, in compound **V**, a C–O bond vibration was observed at ∼1100 cm^–1^, which is absent in the other compounds. Other characteristic FT-IR bands include ∼2950 cm^–1^ (C–H stretching) and ∼1500 cm^–1^ (C=C stretching), which were observed in all the compounds.

The ^1^H NMR spectra of imines **I–V** displayed characteristic singlets for the azomethine proton (CH=N) at 8.32, 8.30, 8.37, 8.36, and 8.33 ppm, respectively. All compounds also displayed signals corresponding to the methine (CH) proton at ∼4.52 ppm and the methyl (CH_3_) proton at ∼1.56 ppm, both adjacent to the stereogenic center. In the spectrum of compound **II**, an additional signal at 2.88 ppm was assigned to the CH_3_ group attached to the aromatic ring. The presence of a chlorine atom induces a deshielding effect on aromatic protons located at the ortho and meta positions, as evidenced by a downfield shift in the corresponding chemical shifts in comparison to unsubstituted systems. Under the experimental conditions employed in this work, no evidence of tautomeric equilibrium was observed, indicating the exclusive presence of a single tautomeric species in solution.

The ^13^C NMR spectra of imines **I–V** revealed azomethine carbon signals in the 157.96–159.15 ppm range. Other relevant signals included ∼24 ppm for the CH_3_ group attached to the chiral carbon and ∼72 ppm for the chiral carbon itself. In compound **V**, the methoxy group displayed characteristic signals at 3.8 and 55 ppm in ^1^H NMR and ^13^C NMR, respectively.

### 3.1 X-ray Crystallographic Analysis

Single crystals of compounds **I–V** were obtained by slow evaporation of CH_2_Cl_2_ at room temperature. The crystallographic data are summarized in Table 1. Single crystal X-ray diffraction (XRD) analyses show that compounds **I–III** crystallize in monoclinic P2_1_ space group, whereas compounds **IV** and **V** adopt the orthorhombic *P*2_1_2_1_2_1_ space group, both of which belong to the Sohncke groups. Structural characterization confirms the presence of the imine (C=N) functional group in all the compounds. The asymmetric unit of compounds **I–III** consists of two independent molecules within the crystal lattice, whereas compounds **IV** and **V** contain four independent molecules per asymmetric unit (Figures S25–S29). The molecular structures of compounds **I–V** were determined via X-ray crystallography, as shown in Figure 1.

**Figure 1:**
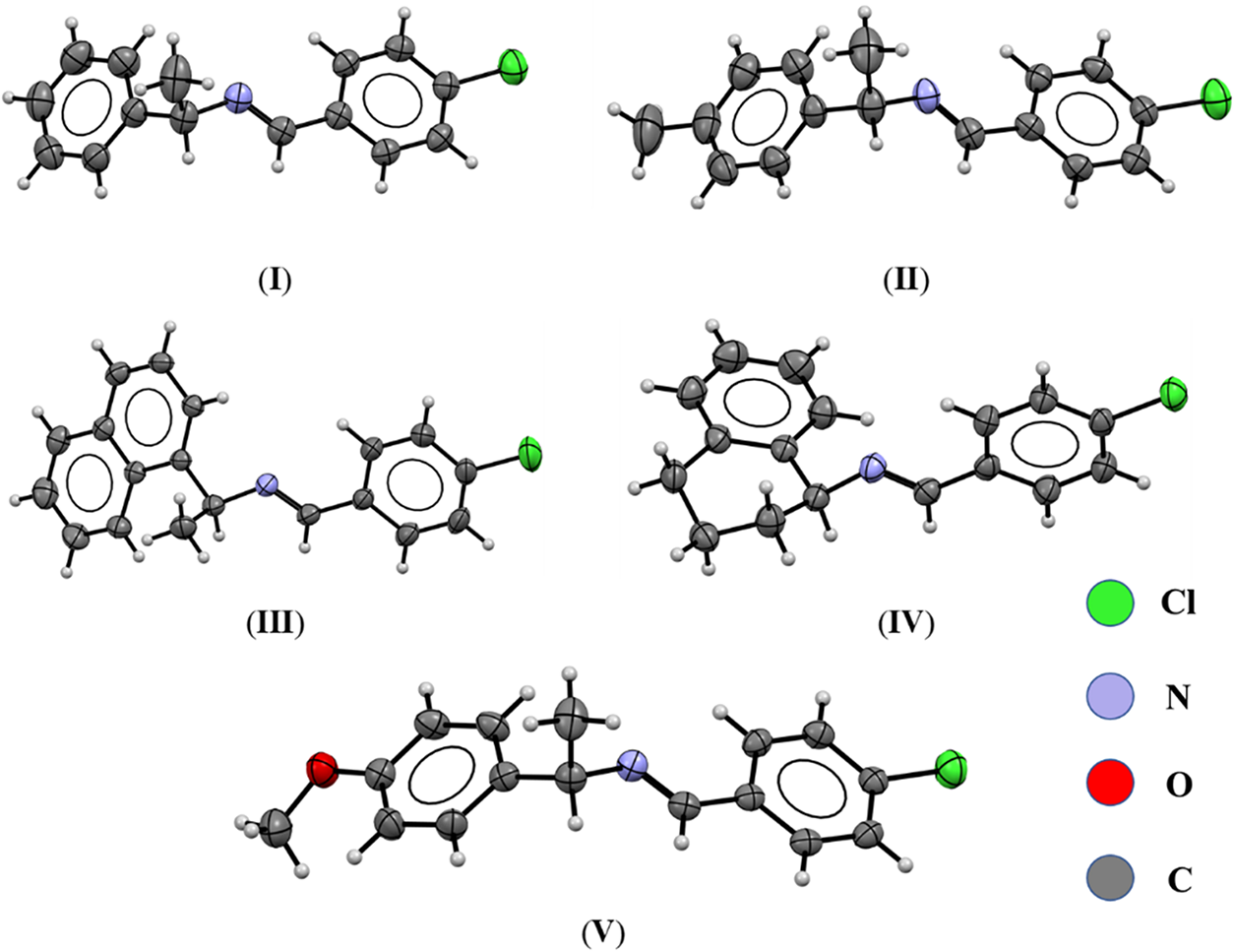
ORTEP diagram of imines **I–V**. Displacement ellipsoids are at the 30% probability level.

The presence of substituents in compounds **I–V** leads to significant deviations from molecular planarity, as evidenced by angles between the chloro-substituted phenyl ring and the adjacent phenyl moiety. The angles between the mean planes of these aromatic rings are as follows: compound **I**, 48.51°; compound **II**, 49.02°; compound **III**, 32.37°, representing the most planar structure, attributed to the stabilizing effect of the naphthyl group on the molecular conformation; compound **IV**, 88.04°, the least planar structure, primarily due to steric hindrance introduced by the cyclohexyl ring; and compound **V**, 45.17°, the second most planar compound, with the methoxy (−OCH_3_) substituent contributing to a reduced torsional angle. Despite differences in molecular planarity, no correlation was identified between molecular conformation and space group.

The analysis of torsion angles across all compounds reveals the steric influence of the substituents. In the ring containing the chlorine atom, the C–C–N–C dihedral angle of the aliphatic chain remains approximately 177 (°) in all compounds.^47^ However, the torsion angle involving the chiral carbon in the aliphatic chain shows an average value of 132° for compounds **I–III** and **V**. The *sp* ^3^ hybridization of the chiral carbon atoms induces deviations from planarity, emphasizing the dominance of steric effects over planar alignment in their crystalline structures.

The crystal structures also confirm two significant bond distances: the imine bond (N=C) with an average length of 1.27–1.30 Å and the C–N single bond with an average length of 1.47 Å. Both values fall within the expected ranges for these types of bonds reported earlier. The key distances and angles of these compounds are summarized in Tables S1–S5.

The centroid-to-centroid distances in the crystal structures of compounds **I–V** were analyzed to identify potential intermolecular π–π stacking interactions between aromatic rings. Compound **V** exhibited the shortest 5.519 Å centroid-to-centroid distance, which exceeds 3.3–3.8 Å value reported in literature, indicating the absence of significant π–π stacking in these structures.

The crystal packing analysis displays differences among compounds **I–V**. Unlike the commonly observed phenyl–phenyl stacking interactions, their stabilization primarily arises from short intermolecular contacts, specifically ∼2.35 Å (C–H···H) and ∼2.89 Å (C–H···C), which play a crucial role in the array of the crystal lattice. The packing arrangement reveals that individual molecules assemble into supramolecular ribbons, which contribute to the three-dimensional architecture.

Additionally, we examined the presence of Cl···Cl interactions in the crystal structures of compounds **I–III** and **V**, and found that the shortest Cl···Cl distance in these compounds exceeds 6 Å. Only compound **IV** shows such an interaction, with a distance of 3.586 Å, which falls within the expected range for twice the van der Waals radius of chlorine (1.76–1.90 Å, corresponding to 3.52–3.80 Å). This distance aligns with typical van der Waals interactions.^48,49^ These interactions have two preferred geometries: Type I, with θ_1_ ≈ θ_2_, and Type II, with θ_1_ ≈ 180° and θ_2_ ≈ 90°. Type I geometry can occur in either cis or trans configurations, while Type II adopts a more defined L-shaped directional arrangement^50,51^ (Figure 2).

**Figure 2:**
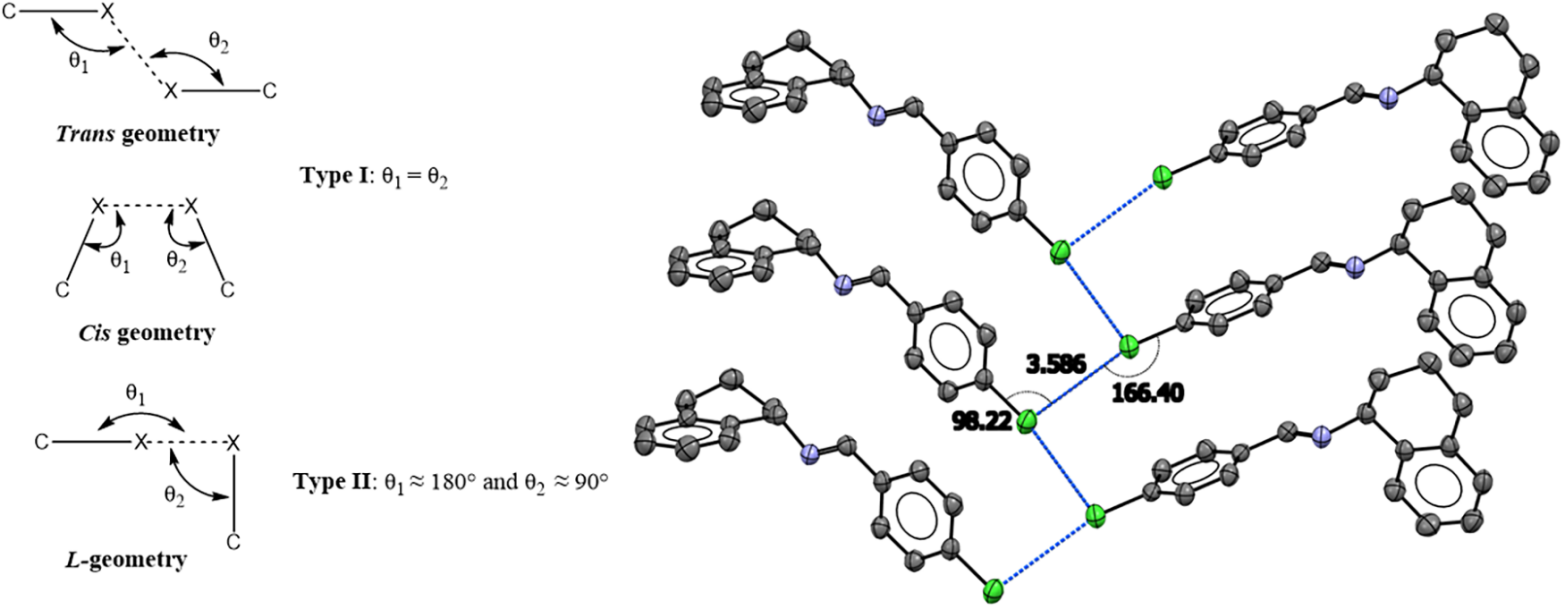
Representation of halogen···halogen contacts: Type *I* (*cis* and *trans* geometries) and Type *II* (*L*-shaped geometry). Packing diagram of compound **IV** illustrating the *L*-shaped Cl···Cl contact, indicated by a dashed line.

In compound **IV**, a Type II geometry is observed, with C–Cl···Cl angles of θ_1_ = 166.40°(8) and θ_2_ = 98.22°(7) (Figure 2). The ipso carbon involved in the C–X···X–C interaction shows sp^2^ hybridization, resulting in an angle slightly larger than the typical 150–160 (°) range. This contrasts with earlier reports indicating that Type II geometries mainly occur when X = I, while Type I geometries are more common for X = Cl, due to the larger size and polarizability of the halogen. In Cl···Cl Type II contacts, the interaction can be viewed as occurring between electrophilic and nucleophilic regions of adjacent halogen atoms.^51–53^

### 3.2 Morphological Characterization

SEM analysis was performed on all samples to complement the structural characterization of the imines and to evaluate the crystal shape, size distribution, and particle orientation. The micrographs (Figure 3) reveal distinct differences in morphology and orientation among imines **I**, **II**, and **IV**, suggesting that the mechanosynthetic route does not afford precise control over the crystal habit of the final products. Image treatment was performed with the ImageJ software, and the discernible grains exhibited average dimensions ranging from approximately 5 to 10 μm. Although these compounds crystallize in related structures, their external morphologies differ considerably.

**Figure 3:**
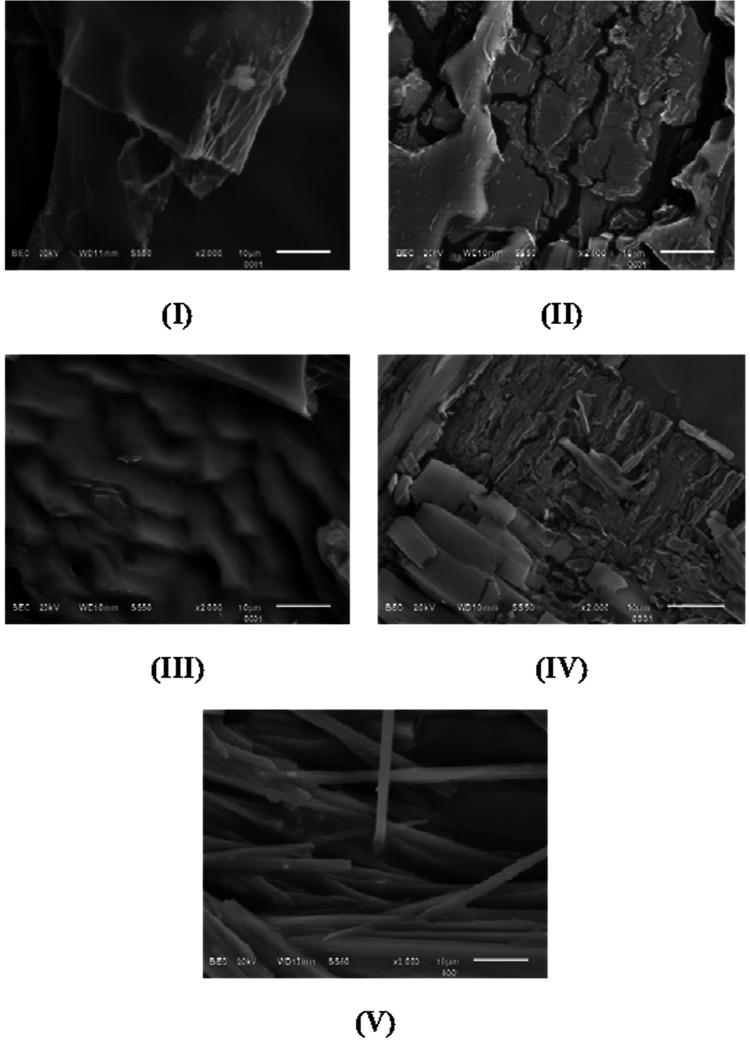
SEM micrographs of chlorinated imines **I–V** showing distinct morphological habits.

The synthesis method, which relies on mechanical energy to induce crystallization, may promote the formation of crystals with structural defects or surface imperfections.^54^ The micrograph of imine **III** shows a lamellar morphology with heterogeneous distribution: some layers appear thinner and elongated, whereas others are wider and shorter, creating a heterogeneous texture. The bar scale indicates that plate thickness varies between 1 and 5 μm. These variations in thickness reflect differences in crystal growth dynamics, likely associated with anisotropic packing forces favored during mechanosynthesis. The surface is relatively rough and undulating, with no clearly defined individual particles observable.

The micrograph of imine **V** displays randomly distributed crystals with a thin, needle-like habit. The acicular crystals measure approximately 1–5 μm in length and 0.2–0.25 μm in diameter, forming elongated structures consistent with anisotropic molecular packing. Such needle-like morphologies are typically associated with directional intermolecular interactions, which favor growth along a preferred crystallographic axis.^55^

The morphological features identified by SEM are in line with the intermolecular packing motifs inferred from single-crystal X-ray diffraction: lamellar growth for imine **III** corresponds to its layered structure of aromatic rings within the crystal lattice.^56^ Needle-like crystals of imine **V** correspond to one-dimensional supramolecular chains stabilized by directional intermolecular interactions related to methoxy substituent. Plate-like and blocky morphologies observed for imines **I** and **II** are consistent with relatively planar packing arrangements, whereas the heterogeneous growth of imine **IV** reflects the steric influence of its substituents that disrupt uniform stacking.^57^ The correlation between microscopic morphology and crystallographic architecture is very strong here, suggesting that the external crystal habit observed by SEM is a direct representation of the molecular packing preferences determined from X-ray analysis.

### 3.3 UV–Vis Absorption

The UV/vis absorption spectra of compounds **I–V** were recorded in acetonitrile solution, as shown in Figures S5, S10, S15, S19, and S24. The data reveal that substituent groups and their positional variations on the benzene rings exert influence on the UV absorption.

All the compounds (**I–V**) exhibit a π → π transition within the 252–254 nm range, with variations in intensity and bandwidth. The broader signal observed in compound **V** is attributed to the inductive effects of the OCH_3_ substituent, which enhances conjugation and stabilizes the excited state. Compound **III**, bearing naphthalene chromophores, exhibits a π → π* transition band at 224 nm, red-shifted as a consequence of increased molecular planarity and enhanced electron delocalization arising from hyperconjugation effects involving the phenyl ring.

X-ray crystal structure analysis confirmed that the lower π → π*transition intensities in compound **IV**, compared to compound **III**, resulted from its significantly less planar molecular structure. The steric effect from the cyclohexyl ring in compound **IV** increases the interplanar angle to 88.04°, the largest among the series, whereas the naphthyl-stabilized planar conformation of compound **III** (interplanar angle: 32.37 (°)) corresponds to stronger π → π* transition intensities. This observation supports the idea that structural changes can modulate the optical and electronic properties of these compounds.

### 3.4 DFT Studies

#### 3.4.1 Geometry Optimization

To assess the reliability of the DFT methods employed, the optimized geometries of imines **I–V** were compared directly with the experimental single-crystal X-ray diffraction data. Selected bond lengths and bond angles were analyzed for all five imines and revealed that both methods, B3LYP and M06–2X, reproduce the experimental single-crystal X-ray data with satisfactory precision and only minor deviations (Tables S1–S5). The optimized bond lengths fall within typical ranges for these types of compounds and are consistent with data reported for related imines.^58^ In the five present crystal structures, the imine C=N double bond averages around 1.256 Å and the adjacent C–N single bond about 1.467 Å, meanwhile B3LYP and M06–2X found similar distances: 1.269 and 1264 Å for C=N, and 1.460 and 1.456 Å for C–N, respectively. This trend is also observed for heavier atoms, such as the average bond length of the C–Cl bond: X-ray diffraction gives an average of 1.738 Å for **I**-**V**, which B3LYP overestimates by about 1.757 Å, while M06–2X gives 1.743 Å, nearly exactly correct. Both functionals work well for most light-atom covalent bonds (such as C–C in aromatic rings), and the differences between calculated and experimental aromatic C–C distances are on the order of 0.01–0.02 Å or less. Similarly, the representative bond angle of the imine group for the imines **I**-**V** falls around 117.47 (°) in the crystal structures whereas B3LYP and M06–2X show 118.33 and 117.91 (°), respectively. For instance, a typical ring angle in one phenyl group is 118.1 (°) and another one is around 121.1 (°) by X-ray and B3LYP and M06–2X showed small differences around 0.5–0.8 (°), confirming that both methods preserve the integrity of the aromatic ring geometry. These small differences of the bond distances and bond angles for all five imines showed that both methods are able to reproduce experimental single-crystal X-ray data with acceptable precision. However, M06–2X consistently yielded lower RMSE and MAE values (Table S6) for most structures, indicating slightly better agreement with experiment. Similarly, M06–2X also yielded more precise parameters, which exhibited higher Pearson correlation coefficients^59–61^ (Table S6 and Figure S30), suggesting it captures the subtle steric and electronic effects of halogenation and aryl substitution. These results emphasize the slightly superior performance of M06–2X due to the high–exact-exchange meta-hybrid DFT method that has been parametrized to handle a wide range of systems with significant electron delocalization, long-range charge-transfer character, and including those with noncovalent interactions. According to these findings, M06–2X calculations were performed to assess the sensitivity of geometrical, electronic, and spectroscopic parameters to the choice of this functional.^62^

#### 3.4.2 Assignments of Vibrational Spectra

The DFT vibration frequencies were determined, scaled (0.952), and the IR spectra were compared with the experimental FTIR spectra for all five imines (Figure S31). Comparative vibrational analyses of imines **I**–**V** confirm spectroscopic signatures attributable to their common Schiff-base and 4-chlorophenyl scaffold: strong C=N stretch (ν­(C=N)) at 1630–1645 cm^–1^, aromatic C–H stretches near 3000 cm^–1^, C=C ring vibrations at 1500 cm^–1^, a C–N stretch in the 1100 cm^–1^ region, and a characteristic C–Cl band at around 760 cm^–1^. Substituents lead to observable perturbations: electron-donating groups increase ν­(C=N) frequencies (naphthyl in **III**: 1646 cm^–1^; methoxy in **V**: 1641 cm^–1^) and intensify aromatic modes. Also, a steric bulk in **IV** causes some frequency/intensity anomalies to its C–Cl vibration due to ring twisting, while methoxy group in **V** introduces a C–O stretch at 1100 cm^–1^. M06–2*X*/6–311+G­(d,p) reproduces all key experimental bands and substituent-driven trends qualitatively, such as elevated ν­(C=N) for **III** and **V**, and steric distortions in **IV**; deviations are systematic, with underestimation of ν­(C=N) frequencies and larger C–Cl discrepancies in **IV** due to steric effects. Nevertheless, the uniform scaling preserves spectral patterns, which allow confident assignment of vibrational modes that confirm electronic effects (conjugation/donation), that collectively explain structure-spectra relationships across the series.

#### 3.4.3 Frontier Molecular Orbital (FMO) Analysis

FMOs analysis can reveal several conceptions such as optoelectronic behaviors, chromophore properties, charge transfer, chemical stabilities, and reactivity.^62^ The HOMO (Highest Occupied Molecular Orbital) and LUMO (Lowest Unoccupied Molecular Orbital) energies have a significant impact on the optical and electronic properties of compounds. The HOMO–LUMO energy gap (Egap) is also important to the stability and reactivity of molecules; the smaller the Egap value means more possibilities for intramolecular charge transfer, and therefore greater reactivities and polarizabilities. Figure 4 shows HOMO–LUMO levels for each of the five imine structures and clearly shows the trends associated with electron delocalization and nucleophilicity. For instance, imines **I**, **II**, and **IV** have a HOMO that is largely localized on the imine nitrogen and adjacent aromatic rings, which indicates only a moderate electron delocalization across the π-system, resulting in well-defined electron-rich regions (on the −C=N– and phenyl groups) rather than extensive conjugation. Therefore, these molecules have stable electron-rich regions without much delocalization which makes them less prone to donate electrons or undergo oxidative processes.

**Figure 4:**
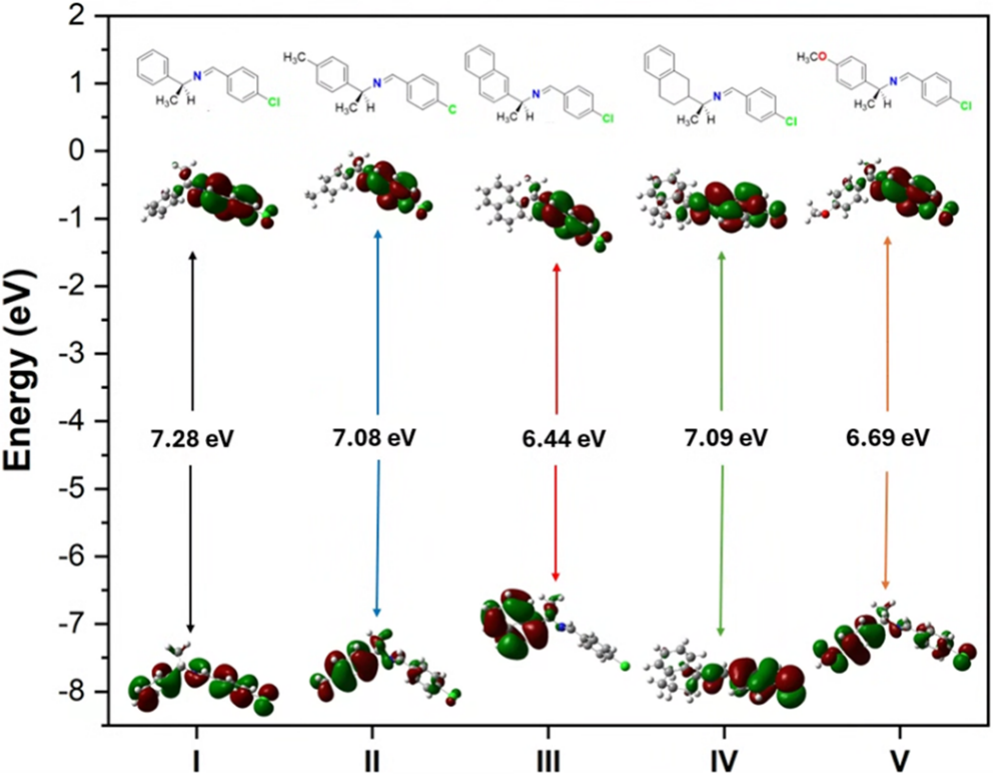
DFT-calculated of HOMO (bottom) and LUMO (top) of imines **I–V** using M06–2*X*/6–311+G­(d,p) level of theory, showing their corresponding energy levels and gaps.

On the other hand, imines **III** and **V** have more delocalized HOMO orbitals across highly conjugated systems (extended π-frameworks involving naphthyl and methoxy substituents), which raises their energy. The HOMO of **III**, extends over the naphthalene moiety in addition to the imine linkage, while in **V** the electron-donating – OCH_3_ group contributes electron density into the aromatic π-system. The higher delocalization of the HOMO also implies higher polarizability and better electronic flexibility that is necessary for materials designed to engage in charge-transfer interactions, such as those designed to interact with transition metals (e.g., metal–organic frameworks [MOFs]) or organic semiconductors used in optoelectronic devices.^63^

The LUMO profiles complete the HOMO characteristics and help to differentiate the chemical potential of imines: Imines **I**, **II**, and **IV** possess a localized and less accessible LUMO orbital located on the imine carbon (and partially on aromatic rings) that results in wider band gaps and lower electrophilic reactivity; whereas imines **III** and **V** show more extended delocalization of LUMO orbitals over the conjugated frameworks.

Molecular planarity plays a critical role in modulating these orbital characteristics. The 4-chlorophenyl substituent in imines **I**, **II**, and **IV** is *ortho* to the imine bond, introducing steric crowding that prevents the aromatic rings from being perfectly coplanar with the C=N bond, reducing π–π overlap between the rings and the imine linkage; thus, orbital shapes are affected by both electronic (donating or withdrawing) and steric effects on planarity. In general, extended planar π-networks (as in **III**) or strong donor groups (as in **V**) result in delocalized, high-energy HOMOs, whereas sterically twisted or electron-withdrawing substituents (**I**, **II**, **IV** with – Cl) render the frontier orbitals more localized and lower in energy.

#### 3.4.4 Global Reactivity Parameters (GRPs) Analysis

Many chemical interactions are determined by the HOMO–LUMO energies such as the global hardness (**η**), global softness (**σ**), global electrophilicity index (**ω**), electronegativity (**χ**), chemical potential (**μ**), electron affinity (AE), and ionization potential (IP), that are considered as GRPs.^43,61^ These descriptors, rooted in conceptual DFT, quantify the propensity of the molecules to donate or accept electrons and their overall chemical stability. Table 2 shows the electronic parameters calculated for compounds **I**-**V**, which offers insight into their different aspects of reactivity, stability, or optical behavior.^64^

**Table 2:** DFT-Calculated Global Reactivity Parameters for Imines I–V at M06-2*X*/6-311+G­(d,p) Level of Theory

parameter	formula	I	II	III	IV	V
*E*_HOMO_ (eV)	---	–8.090	–7.857	–7.294	–7.899	–7.451
*E*_LUMO_ (eV)	---	–0.814	–0.772	–0.852	–0.807	–0.763
*E*nergy gap (*E*_gap_, eV)	*E*_LUMO_ – *E*_HOMO_	7.277	7.085	6.442	7.092	6.688
ionization potential (IP, eV)	-*E*_HOMO_	8.090	7.857	7.294	7.899	7.451
electron affinity (EA, eV)	-*E*_LUMO_	0.814	0.772	0.852	0.807	0.763
hardness (η, eV)	$\frac{\left(\mathrm{IP}-\mathrm{EA}\right)}{2}$	3.638	3.542	3.221	3.546	3.344
chemical potential (μ, eV)	$-\frac{\left(\mathrm{IP}+\mathrm{EA}\right)}{2}$	–4.452	–4.315	–4.073	–4.353	–4.107
electronegativity (χ, eV)	$\frac{\left(\mathrm{IP}+\mathrm{EA}\right)}{2}$	4.452	4.315	4.073	4.353	4.107
electrophilicity index (ω, eV)	$\frac{\mu^{2}}{2\eta }$	2.724	2.628	2.575	2.672	2.522
softness (σ, eV^–1^)	$\frac{1}{\left(\mathrm{IP}-\mathrm{EA}\right)}$	0.137	0.141	0.155	0.141	0.150
polarizability (α, Å^3^)	$\frac{\alpha_{\mathrm{XX}}+\alpha_{\mathrm{YY}}+\alpha_{\mathrm{ZZ}}}{3}$	22.519	23.074	30.279	24.207	23.268

GRPs derived from DFT calculations delineate two distinct electronic classes among imines **I**–**V**. The first class (**I, II**, and **IV**) show high ionization potentials, large HOMO–LUMO Egap, and increased chemical hardness (localized electron density), kinetic stability, resistance to redox processes, low polarizability, and high electronegativity (limited delocalization of electrons, minimal electronic perturbation), which make them suitable for applications that need oxidative stability and minimally photochemically reactive.

In contrast, the second class (**III** and **V**) show a reduced IP, smaller HOMO–LUMO Egap, and lower hardness with improved redox activity, higher chemical potential, and softness for intramolecular charge transfer and electronic excitations. Thus, their longer π-conjugation in **III** (naphthyl group) and electron-donation in **V** (*p*-OCH_3_) lower ionization barriers and enhance optical responsiveness to make these compounds candidates with high polarizability for effective light-matter interaction and charge-transfer processes.

Collectively, substituents dictate reactivity: halogenated/alkyl groups (**I**, **II**, **IV**) promote stability, while π-extended or electron-donating groups (**III**, **V**) enhance electronic delocalization and functional versatility.

#### 3.4.5 Molecular Electrostatic Potential (MEP) Surface

The MEP maps for the imines were calculated by M06–2*X*/6311G+(d,p) level of theory to predict the chemical reactivity, electron donor and acceptor sites of the studied compounds.^64^ Figure 5, shows MEP surfaces of **I**-**V** where the highest positive potential region, colored in dark blue, is distributed over the carbon atom next to the imine bond; while the highest electronegative potential region, colored in red, is centered around the aromatic group and the nitrogen atom for **I**, **II**, **III** and **V** and also localized around the oxygen atom in **V**. According to the MEP surface, it can be said that all these imines have electron-rich nitrogen atoms and are electron-deficient at the imine carbon as required for nucleophilic attack. Along with data in Section 2.4.1, compounds **I**, **II**, and **IV** have lower melting points (Mp), that correlate the poor electronic properties (weakly conjugated lower polarizability, higher hardness, lower electrophilicity, and higher bandgap), discussed in the previous section. In contrast, imines **III**, and **V** have better electronic properties (highly conjugated π-systems, higher localization of density at substituent naphthyl and methoxy groups, higher polarizability, lower hardness, and lower bandgap) which correlate the higher Mp due to the intermolecular interactions.

**Figure 5:**
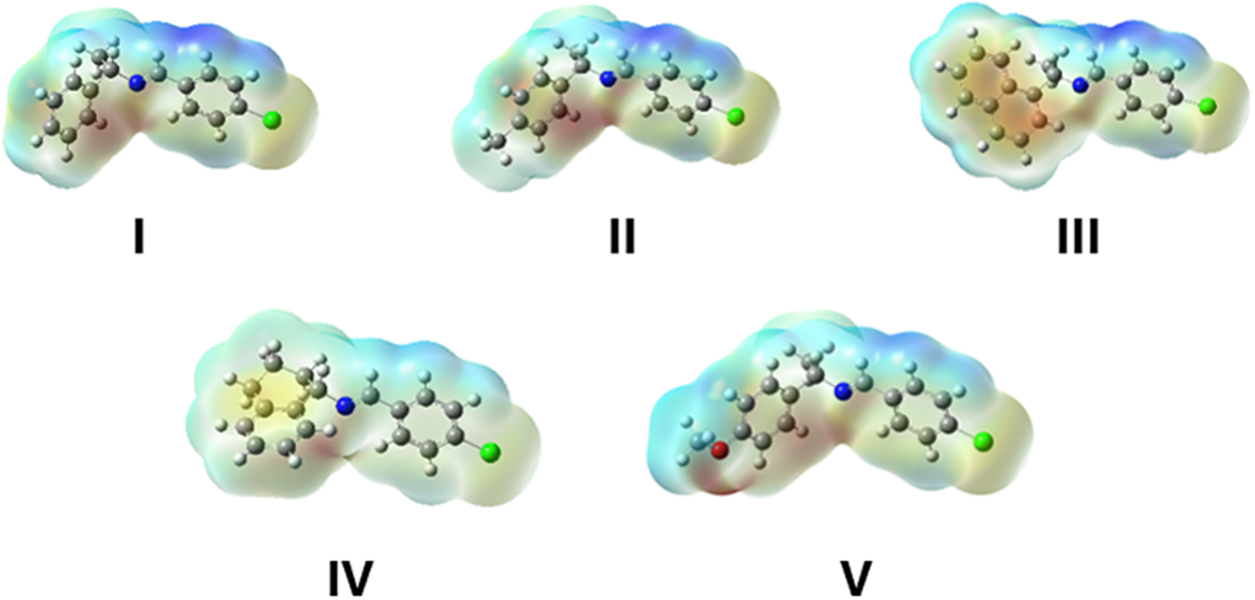
MEP surfaces calculated by M06–2*X*/6–311+G­(d,) level of theory for **I–V**.

#### 3.4.6 Natural Bond Orbital (NBO) Analysis

The NBO analysis provides insights into the maximum electron density distribution among the orbitals.^59^ The stabilization energy *E* ^(2)^ related to delocalization from i (donor) to j (acceptor) is given by

$$E^{\left(2\right)}=-q_{i}\frac{{\left(F_{\mathrm{ij}}\right)}^{2}}{\varepsilon_{j}-\varepsilon_{i}}$$

where *q*_*i*_ is the donor orbital occupancy, ε*_i_*, ε_*j*_ are diagonal elements (orbital energies) and *F*_*ij*_ is the off-diagonal NBO Fock matrix element.^58^ The interaction stabilization energy (*E* ^(2)^) and donor and acceptor electron orbitals are tabulated (Tables S7–S11). The electron densities of the donor and acceptor NBO orbital are denoted by ED/e. Tables S3–S11 include three forms of transitions such as π → π*, LP → σ*, and LP → π* where E^(2)^ greater than 8 kcal/mol have been chosen for interactions between electron donors and acceptors as intensive.

NBO analysis reveals a rich network of intramolecular donor–acceptor (Lewis to non-Lewis) interactions in the chiral imines **I**–**V**. All imines show strong intraring delocalization (*E* ^(2)^ around 25–31 kcal/mol), which is typical for aromatic resonance, but more importantly, the imine π bond itself participates in conjugative interactions with adjacent phenyl rings. In each case, the aromatic π orbitals donate into the imine π*, π­(C–C)→π*­(N–C), with stabilization energies of 17–18 kcal/mol, while the imine π donates back into aromatic π* orbitals, π­(N–C)→π*­(C–C), with slightly less stability (9–10 kcal/mol), reflecting that the phenyl ring bonded to the imine carbon is a more effective electronic donor toward C=N than the aniline ring attached to nitrogen (Tables S7–S11). This delocalization is affected by the nature of substituents, and the extent of conjugation depends on it: imines **III** (naphthyl) and **V** (methoxy-substituted phenyl) have longer π networks correlating with their experimentally narrower HOMO–LUMO gaps and higher polarizability.

Electron distribution is further modulated by lone-pair donations into aromatic π systems, where the chloro substituents in all five imines contribute modest n­(Cl)→π* interactions (*E*(2) ≈ 15–16 kcal/mol), suggesting weak resonance donation that slightly enriches the aromatic rings. Imine **V** exhibits a strong n­(O)→π* interaction from its methoxy group (*E* ^(2)^ = 35.2 kcal/mol, Table S11), significantly increasing electron density on the ring and accounting for its high HOMO energy and nucleophilic character, while imine **IV** shows substantial delocalization of its imine group into the aromatic system but is sterically distorted by the ortho chlorine, limiting full conjugation across the framework. Other hyperconjugative effects were also noted, remarkably n­(N)→σ­(C–H)* interactions (E^(2)^ = 14.29 kcal/mol, Table S10), that stabilize the imine conformation and support the planar geometry needed for conjugation.

Collectively, these donor–acceptor interactions account for the different electronic behaviors observed between the imines **III** and **V** with stronger substituent-driven conjugation resulting in more delocalized frontier orbitals, smaller HOMO–LUMO gaps, and larger softness that are consistent with higher reactivity and polarizability versus imines **I**, **II**, and **IV** which rely predominantly on the baseline aromatic and imine conjugation to yield larger gaps and lower hardness. Hence, NBO results not only offer a molecular-level picture of charge transfer and delocalization pathways but also directly rationalize trends in electronic structure, reactivity descriptors, and electrostatic potential surfaces derived from DFT calculations.

#### 3.4.7 UV–Vis and Electronic Circular Dichroism (ECD) Spectra

UV–Vis spectra (Figure 6a) of **I**–**V** obtained at the M06–2*X*/6–311+G­(d,p) level (including solvent effects) show that the optical properties are modulated by substituents. All five of the imines show two major absorption bands: a high-energy π–π* transition in the far-UV and a lower energy band in the near-UV. The intense π→π* excitation (localized primarily on the C=N chromophore and adjacent aromatics) is observed for **I**, **II**, **IV**, and **V** at 190–200 nm due to their some extent less conjugated systems. However, imine **III** shows a markedly red-shifted π–π* band around 220 nm because of its extended aromatic conjugation (through the naphthyl ring) and greater planarity (increasing the conjugation), reducing transition energies such that π–π* and n−π* transitions combine into a broad continuum. A second, weaker absorption at about 250–260 nm is assigned to n→π* transitions involving heteroatom lone pairs (imine nitrogen or substituents such as -Cl, -OCH_3_) interacting with the π* system.

**Figure 6:**
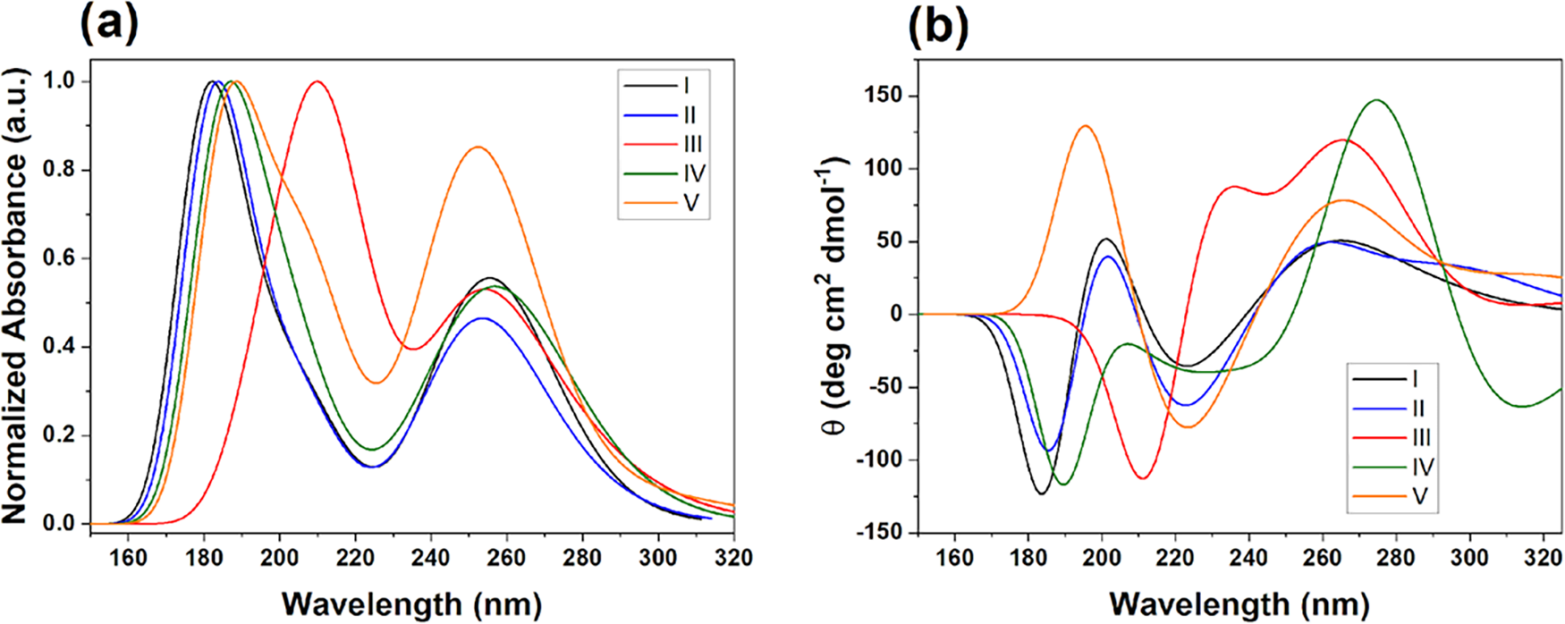
Theoretically calculated (a) UV absorption, and (b) ECD spectra using acetonitrile as solvent of the imine compounds **I–V** at TD-DFT M06–2*X*/6–311G+(d,p) level.

The near-UV band is always present throughout the series but its position and intensity varies according to substituent effects: for example, in imines **I**, **II** and **IV** (each bearing an ortho-chloro substituent) the n→π* peak occurs at 252, 257, and 260 nm, whereas imine **V** (*p*-OCH_3_) shows a similar band at 258 nm, which is broadened and enhanced by inductive donor effects. The electron-donating character of the 4-methylphenyl group in **II** causes only a minor red-shift relative to **I**. The fact that these two types of transition are found across all compounds suggests a common underlying electronic structure motif, with substituents affecting the energy and oscillator strength of similar π–π* and n−π* excitations rather than introducing fundamentally different transitions. Imine **IV** (the phenylcyclohexyl) extends out into longer wavelengths with an extremely broad maximum around 260 nm (along with its normal far-UV band), because its geometry is significantly twisted by the induced torsion and reduced planarity of this bulky group, which allows some intramolecular charge-transfer character. Imine **V** (*p*-OCH_3_) has a sharp peak at high energy near 197 nm that is followed by broadening at longer wavelengths with an intensity somewhat greater than imine **III** but not extending into the low-energy region.

The TD-DFT-calculated spectra agree well with experiment (Figure S32), reproducing band positions and relative intensities within minor offsets for imines **I**–**V**, which captures observed substituent-dependent trends. Imine **III** has the most red-shifted profile, consistent with its experimental spectrum having the longest-wavelength onset; and the very broadened 250–260 nm band in the experimental spectrum of **V** (owing to the conjugative effect of the *p*-OCH_3_ group) is also reproduced by the TD-DFT calculation.

The calculated ECD spectra at M06–2*X*/6–311+G­(d,p) level for the imines **I**–**V** exhibit Cotton effects that reflect their electronic structure as well as substituent effects. Imines **I** and **II** show relatively simple spectral profiles, with weak bisignate signals in the 220–260 nm region arising from n→π* transitions on the imine group and adjacent phenyl rings. A *p*-methyl substituent in imine **II** causes a slight red-shift and slightly stronger signal than that of imine **I**, consistent with its marginal electron-donating effect. On the other hand, imine **III** has the largest and most red-shifted Cotton effects (230 nm to near-UV) owing to its naphthyl substituent that favors extended π-conjugation, which decreases excitation energies and increases chiroptical response because of more efficient coupling between the extended π-system and the inherent chirality of the imine scaffold. Imine **IV** has a broadened ECD profile with weak positive and negative bands in the 240–280 nm range due to steric disruption and loss of planarity caused by the cyclohexyl substituent and *ortho*-chloro substitution. Meanwhile, imine **V** with *p*-OCH_3_ group has an intense negative Cotton effect around 250 nm that is directly related to the increase in n→π* mixing and electronic polarization from the electron-donating substituent.

#### 3.4.8 Noncovalent Interaction (NCI) Analysis

The NCI analysis in Figure 7 shows that each imine (**I**–**V**) has a specific intramolecular interaction profile at the M06–2*X*/6–311G+(d,p) level. **I** and **II**, have broad spikes near zero with only faint blue-green patches in their 3D reduced density gradient (RDG) isosurfaces, indicating weak dispersion forces (mild attractive contacts like C–H···π or C–H···Cl hydrogen-bond-like interactions) and no steric repulsion. Meanwhile **III**, bearing an extended naphthalene ring, has the largest stabilizing interaction in the series with a distinct negative-sign­(λ_2_)­ρ spike and a 3D RDG isosurface revealing extensive green and blue-green regions sandwiched between its nearly coplanar aromatic rings, confirming robust intramolecular π-stacking stabilization with negligible steric hindrance. Imine **IV**, which contains a bulky cyclohexyl substituent that disrupts conjugation, has no significant negative spike in its NCI scatter plot but exhibits a slight positive (red) shoulder instead and has red lobes of steric clash between the cyclohexyl and phenyl rings in the isosurface, indicating very weak intramolecular attractive contacts only destabilizing strain due to enforced twist.

**Figure 7:**
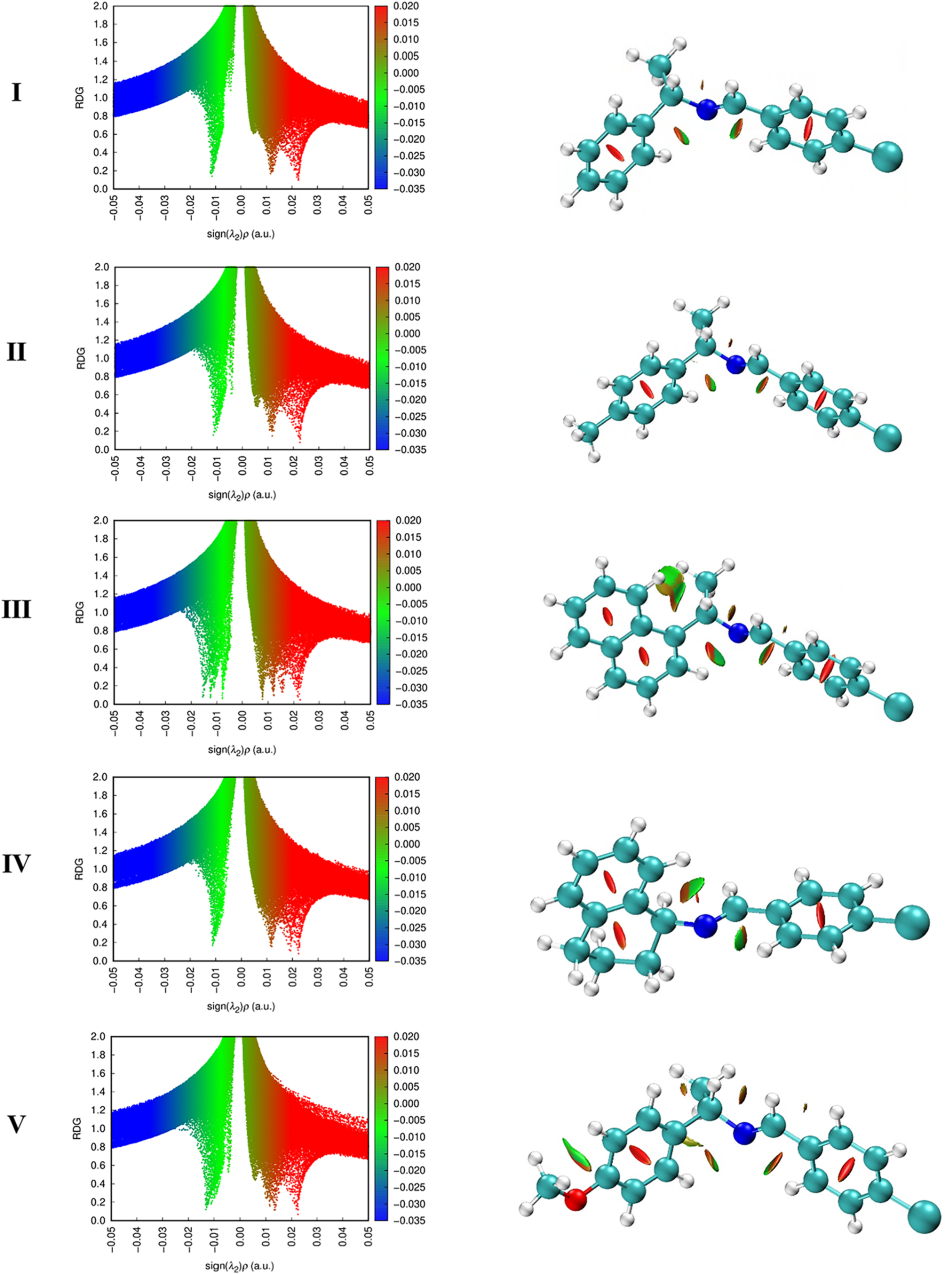
RDG vs sign­(λ_2_)­ρ scatter plots (left) and real-space RDG isosurfaces (right) for the intramolecular NCIs in imines **I–V**, obtained from M06–2*X*/6–311+G­(d,p) electron densities.

The molecular structure and stability of these imines **I**–**V** are directly relevant to the NCI findings. The greater internal π–π attractions in **III** (and to a lesser extent, **V**), which promote more planar, rigid structures, for example, inter-ring dihedral of 32 (°) versus 88 (°) for sterically hindered **IV**, provide further intramolecular stabilization that may account for its greater rigidity and the large red-shift of its electronic transitions. Imine **V** also benefits from increased conjugation and polarizability with an electron-donating methoxy group, although to a lesser extent than the polycyclic imine **III**. In contrast, the bulky aliphatic substituent on imine **IV** prevents coplanarity and precludes any meaningful π-stacking, adopting a twisted geometry (essentially orthogonal rings) with no intramolecular stabilization, consistent with NBO analysis and DFT-derived electronic properties (larger Egap and higher η). Thus, **I**, **II**, and **IV** have greater thermodynamic stability (but lower polarizability), while **III** and **V** exhibit more delocalized electronic structures, in which intramolecular dispersive interactions play an important role. The NBO data are consistent with the NCI results and indicate that there is no evidence of a significant intramolecular charge-transfer or halogen–bond interaction in these molecules (such as any substantial donor→acceptor orbital overlap between the chlorine substituents), suggesting that the chlorine acts only as an inductive/electrostatic effect. Therefore, intramolecular NCIs (or the lack thereof) determine if the molecule adopts a favored planar conformation or if is forced into a twisted, strained one, which affects its internal stability and flexibility.

The overall picture shows that the NCI-derived insights into hydrogen-bonding, van der Waals, and steric effects are in excellent agreement with both the electronic structure calculations and experimental spectral data: stabilizing NCIs (such as π–π and C–H···X contacts) prefer planar, conjugated structures that maximize UV–vis absorption and ECD signals. Conversely, destabilizing or absent NCIs yield twisted geometries with reduced conjugation and correspondingly weaker or higher-energy spectroscopic features. The combination of NCI-derived insights into hydrogen-bonding, van der Waals, and steric effects with electronic structure calculations and experimental spectral data provides a comprehensive picture showing that subtle intramolecular noncovalent forces can determine the conformation and photophysical properties of chiral Schiff bases.

#### 3.4.9 QTAIM Analysis

QTAIM analysis offers an approach to studying chemical bonding through examination of bond paths and critical points such as bond critical points (BCP), which is a useful indicator of interactions between two molecular systems (+3, – 1) and specifically describes the hydrogen bond in terms of electron density (ρ) and its Laplacian (∇^2^ρ) at BCPs. QTAIM analysis also identifies BCPs linking atoms and provides topological data to classify interactions.^61,64^

A QTAIM analysis of **IV** revealed two (3,–1) BCPs between pairs of chlorine atoms (Cl­(A) and Cl­(B)) shown in Figure 8 that indicate very weak Cl···Cl contacts (ρ ≈ 0.00536 au; ∇^2^ρ ≈ + 0.0203 au) which are barely discernible from the RDG scatter plot and a slight attractive character (light blue-green NCI color, Figure 7, **IV**). However, the effect is so weak that there appears to be no deviation in the RDG plot of zero. These QTAIM attributes unambiguously establish that the Cl···Cl contact is a weak halogen–halogen interaction (as discussed in Section 3.1 for Type II halogen interactions) rather than a true bond. Here, the bond path is present but the very small electron density and positive Laplacian clearly classify it as a weak dispersion attraction, which QTAIM reveals that imine **IV** possesses two exceptionally weak halogen···halogen ″bonds″ in addition to the strong steric repulsion already identified.

**Figure 8:**
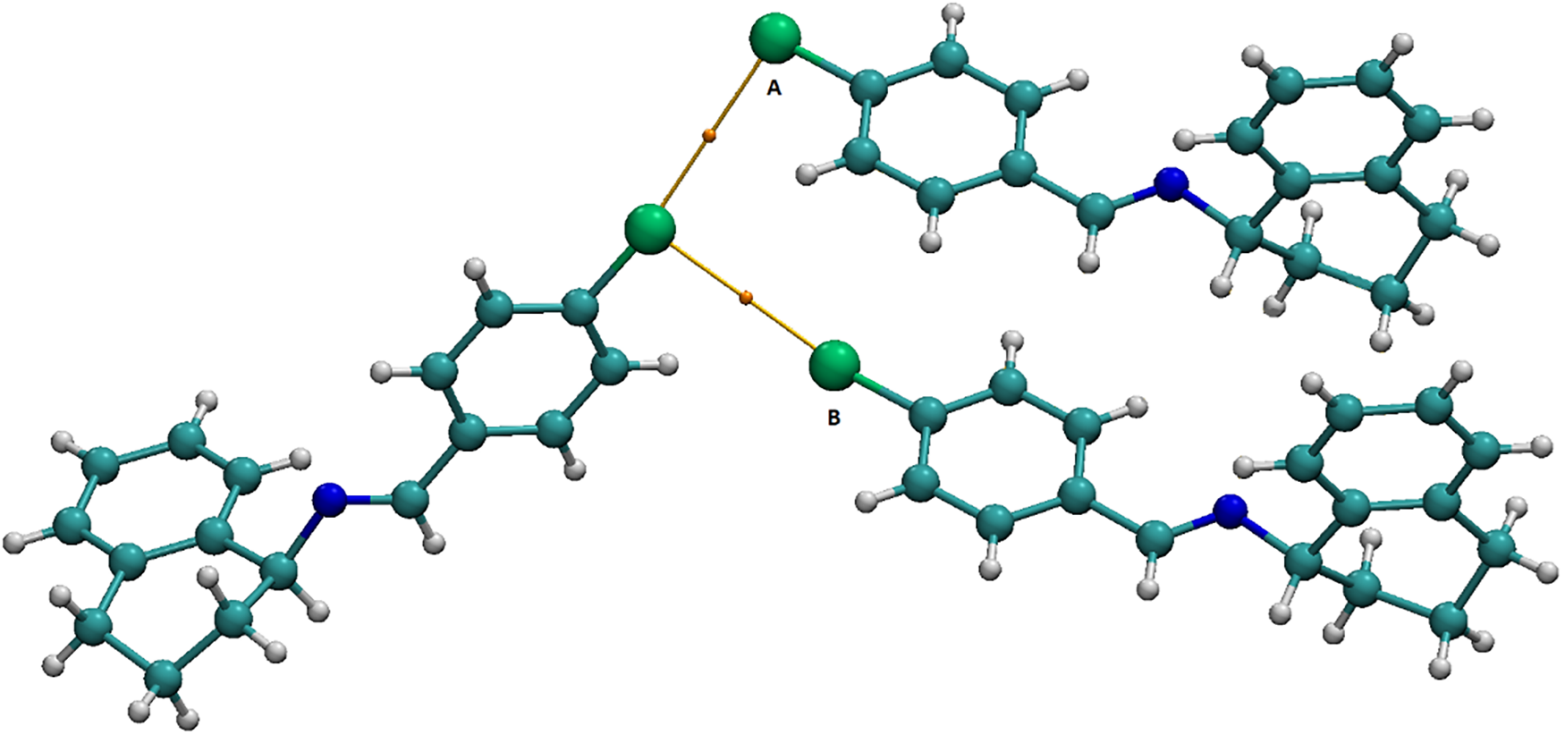
QTAIM molecular graph of imine **IV** showing the two BCPs (red spheres) identified between chlorine atoms.

In NBO analysis, any donor–acceptor charge transfer between these two chlorines would be expected to be very small, given that the lone pair on one Cl has negligible overlap with the antibonding orbitals of the other, leading to a very weak *E* ^(2)^ (<1 kJ/mol), which is consistent with the QTAIM picture of nearly nonbonding interaction. Collectively, the data present a clear picture of imine **IV** that is stabilized primarily by dispersion/steric packing, with only very weak Cl···Cl contacts of van der Waals character.^58^

## 4 Conclusions

In this study, we have theoretically and experimentally investigated five chiral imines synthesized via an environmentally friendly solvent-free strategy. Characterization analysis of imines **I**-**V** shows how substituent groups affect their structural, electronic, and optical properties. Single-crystal X-ray diffraction confirmed the presence of the imine (C=N) functional group and revealed that naphthalene substituents increase molecular planarity, while noncovalent interactions stabilize the supramolecular architecture. According to NMR data, electron-withdrawing groups like chloro cause nearby nuclei to shift downfield due to resonance effects. Additionally, UV–Vis spectra indicated that groups such as – OCH_3_ cause red shifts in absorption peaks through inductive effects, highlighting the role of substitution in tuning optical behavior.

DFT geometry optimizations were found in excellent agreement with crystallographic data, with low RMSD and MAE values and good Pearson correlation coefficients. A slightly better statistical correlation was observed for bond distances and angles using M06–2*X*/6–311G+(d,p) compared to B3LYP/6–311G+(d,p), validating the robustness of the computational models used and highlighting their predictive power for structurally related systems (**I**-**V**). Spectroscopic properties predicted by DFT calculations were found to be in close agreement with the experimental IR, and UV–Vis spectra. M06–2X demonstrated superior performance in reproducing excitation energies, absorption maxima, and Cotton effects associated with charge-transfer transitions. This highlights the importance of employing functionals designed to account for long-range and noncovalent interactions when evaluating optoelectronic properties in conjugated organic systems.

Based on frontier molecular orbital and global reactivity parameter analysis, the electronic regimes of imines **I**, **II**, and **IV**, with localized HOMOs and larger energy gaps, are more electronically stable and less reactive, while those of imines **III** and **V**, which are stabilized by extensive π-delocalization, have narrower gaps, increased softness, and higher polarizability. Their calculated optical properties were consistent with the experimental, where the absorptions of **III** and **V** were red-shifted relative to **I**, **II**, and **IV**, and their Cotton effects were stronger, indicative of a greater charge-transfer character.

NBO and NCI analyses indicated that electronic stabilization is dominated by intramolecular charge transfer from lone pairs to antibonding π* orbitals, especially for the more conjugated derivatives. Weak noncovalent contacts such as dispersive interactions and C–H···π associations were observed throughout the series, and QTAIM analysis revealed two Cl···Cl intermolecular contacts only in imine **IV**. In addition to providing insight into supramolecular organization, these weak and closed-shell halogen interactions could also be involved in packing-dependent optical responses.

Overall, this multidisciplinary study shows that combining theoretical methods with experimental spectroscopy and crystallography provides a consistent and complete picture of how electronic delocalization, reactivity, and chiroptical behavior are affected by substituents in chiral imines and can serve as design principles for the further development of optoelectronic and functional chiral materials.

## Supplementary Material



## Notes

Crystallographic distance and angles; molecular packing representation; crystallographic experimental information; nuclear magnetic resonance spectra for the compounds (^1^H, and ^13^C); FT-IR spectra; MS spectra; and computational graphics (PDF)
