# Supplementary material for: Solvent-Free Synthesis of Chiral Substituted N‑Benzylideneanilines Imines: X‑ray Structure, and DFT Study
Source: ACS Omega. 2025 Oct 22;10(43):51170–85. doi: 10.1021/acsomega.5c06111 (PMC12593969; doi:10.1021/acsomega.5c06111)
Supplement: Supplementary file 1 [file ao5c06111_si_001.pdf]

SUPPLEMENTARY INFORMATION

Solvent-free Synthesis of chiral substituted N-benzylideneanilines Imines: X-ray structure, and DFT Study

Guadalupe Hernández Téllez[1,], José A. Reyes-Avendaño[2], José M. Bravo-Arredondo[3,4], Gloria E. Moreno Morales[1], Pankaj Sharma[5], Claudia P. Villamizar[5], Angel Mendoza[6] and Bertin Anzaldo[1,*]*

1. Lab. Síntesis de Complejos. Fac. Ciencias Químicas, Benemérita Universidad Autónoma de Puebla, Edif. FCQ-6, C.U. Av. San Claudio y Blvd. 14 Sur, Col. San Manuel, C.P. 72592, Puebla, Pue. México
2. Benemérita Universidad Autónoma de Puebla, Facultad de Ingeniería Química, Apartado Postal J-48, C. P. 72570 Puebla, Puebla, México.
3. Benemérita Universidad Autónoma de Puebla, Posgrado en Dispositivos Semiconductores, Prolongación 14 Sur, IC5, Puebla, Puebla, MX 72570
4. Universidad Autónoma de Tlaxcala, Facultad de Ciencias Básicas, Ingeniería y Tecnología, C.P. 90401 Apizaco, Tlax., México.
5. Instituto de Química-UNAM, Circuito exterior, C.U. Coyoacán, C.P. 04510, México, CDMX
6. Centro de Química del Instituto de Ciencias, Benemérita Universidad Autónoma de Puebla, 18 Sur y Av. San Claudio, Col. San Manuel, Puebla 72570, México

1. **Spectroscopy ^{1}H RMN, ^{13}C, IR-ATR, UV-Vis and mass spectrometry spectra for compound I-V**

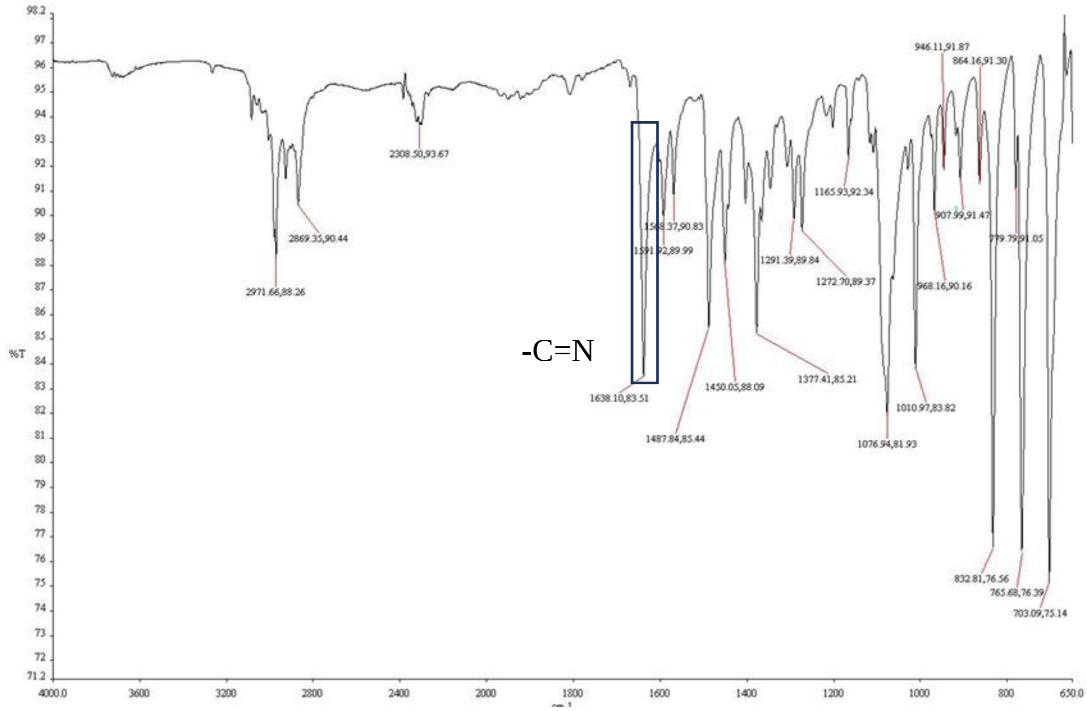

Figure S1. FT-IR spectrum of imine **I**

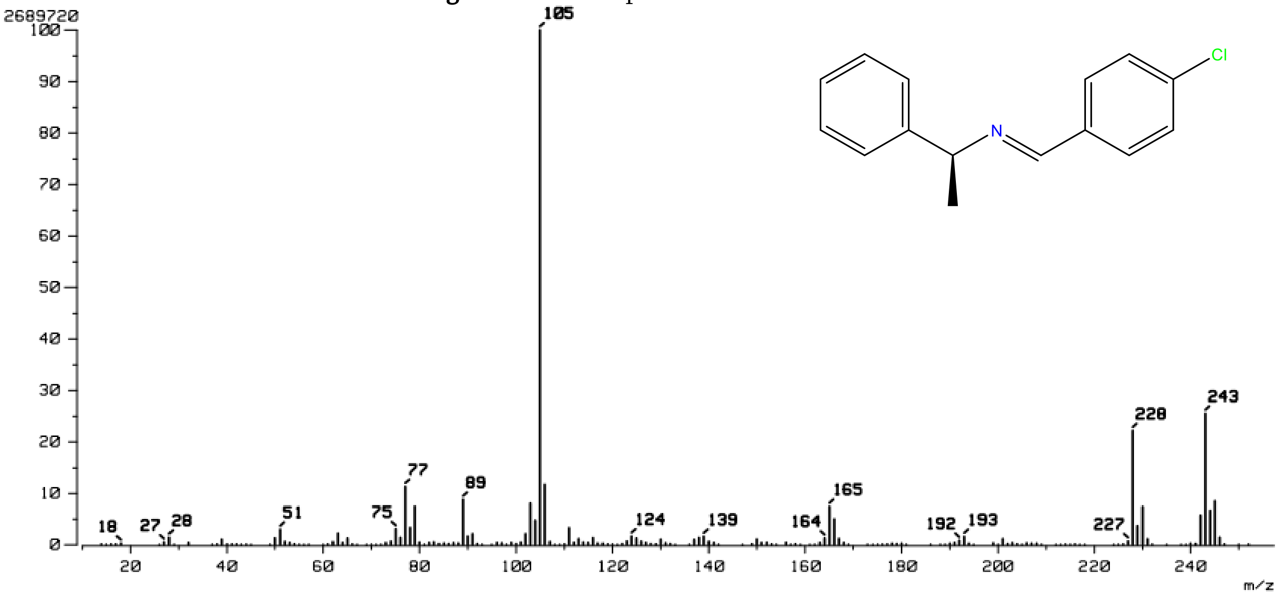

Figure S2. EI⁺ mass spectrum of imine **I**

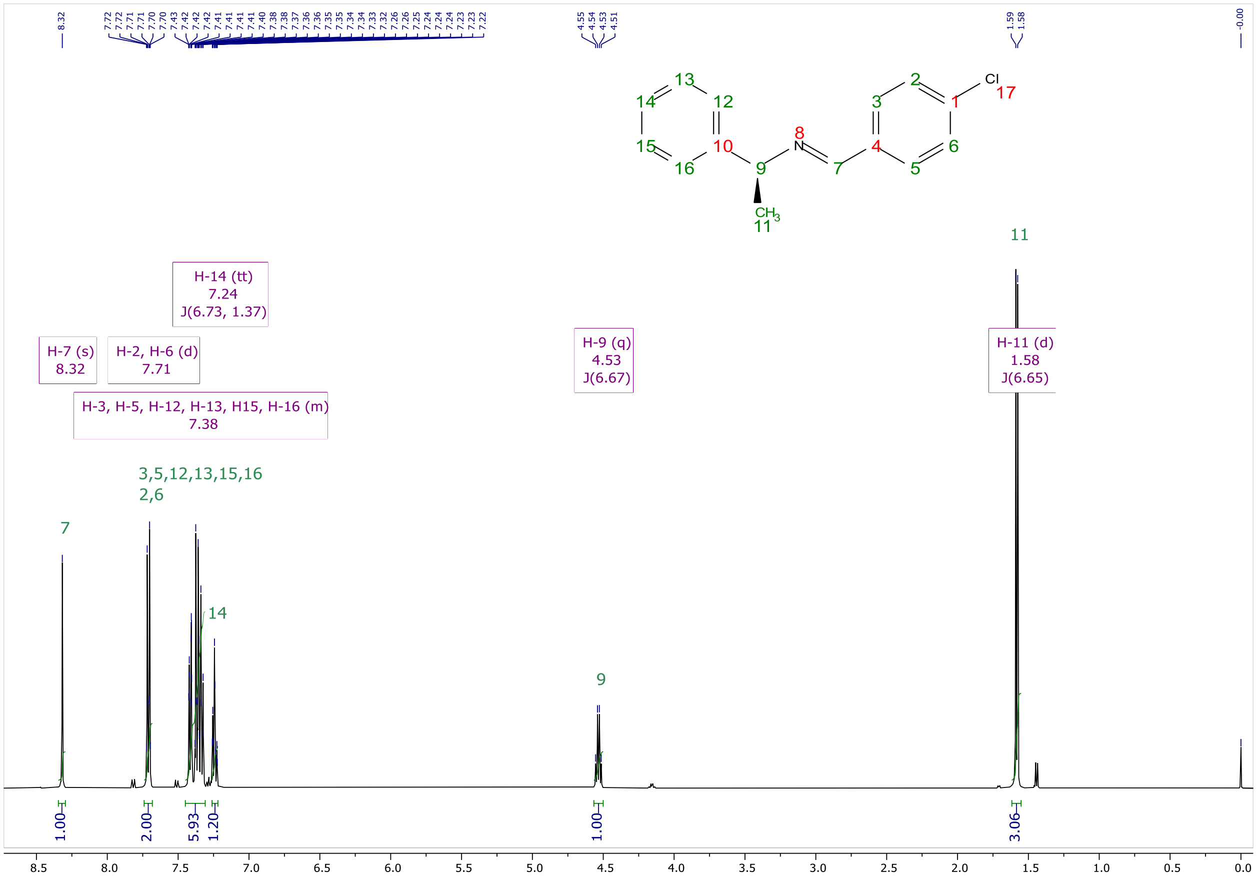

Figure S3. ^{1}H-NMR(500 MHz, $CDCl_3$) spectrum of imine **I**

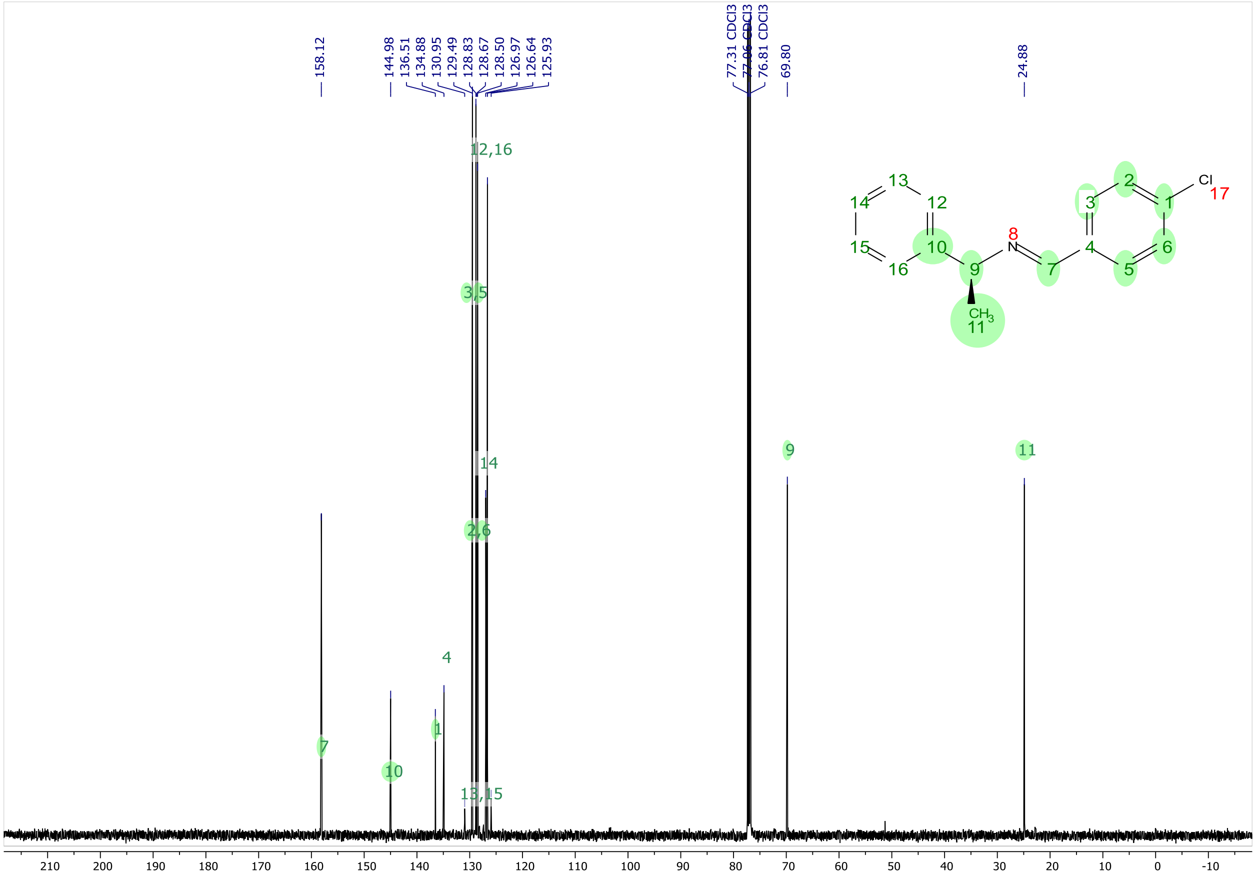

Figure S4. ^{13}C-NMR (125.5 MHz, $CDCl_3$) spectrum of imine **I**

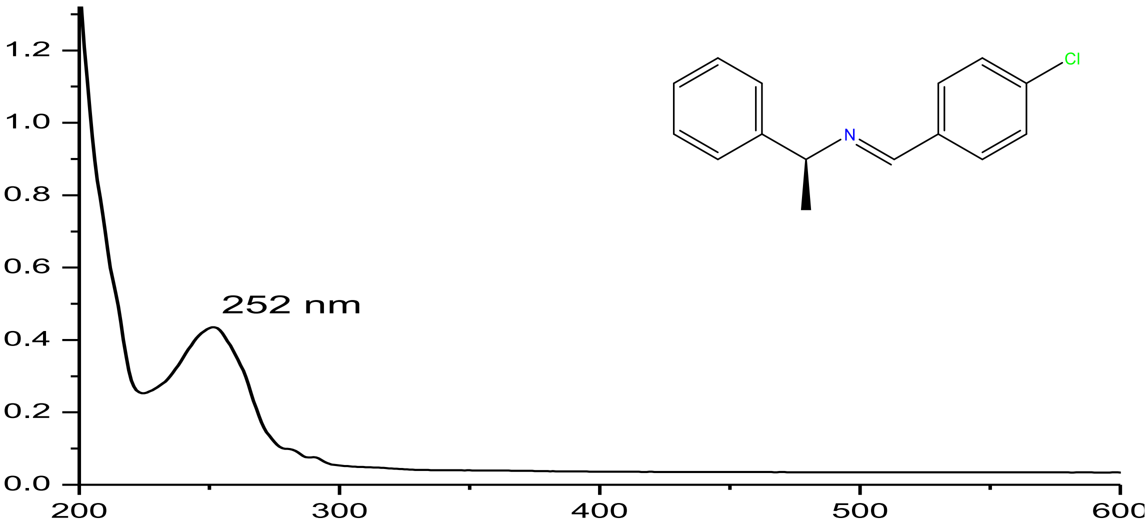

Figure S5. UV-Vis spectra (acetonitrile) of imine **I**

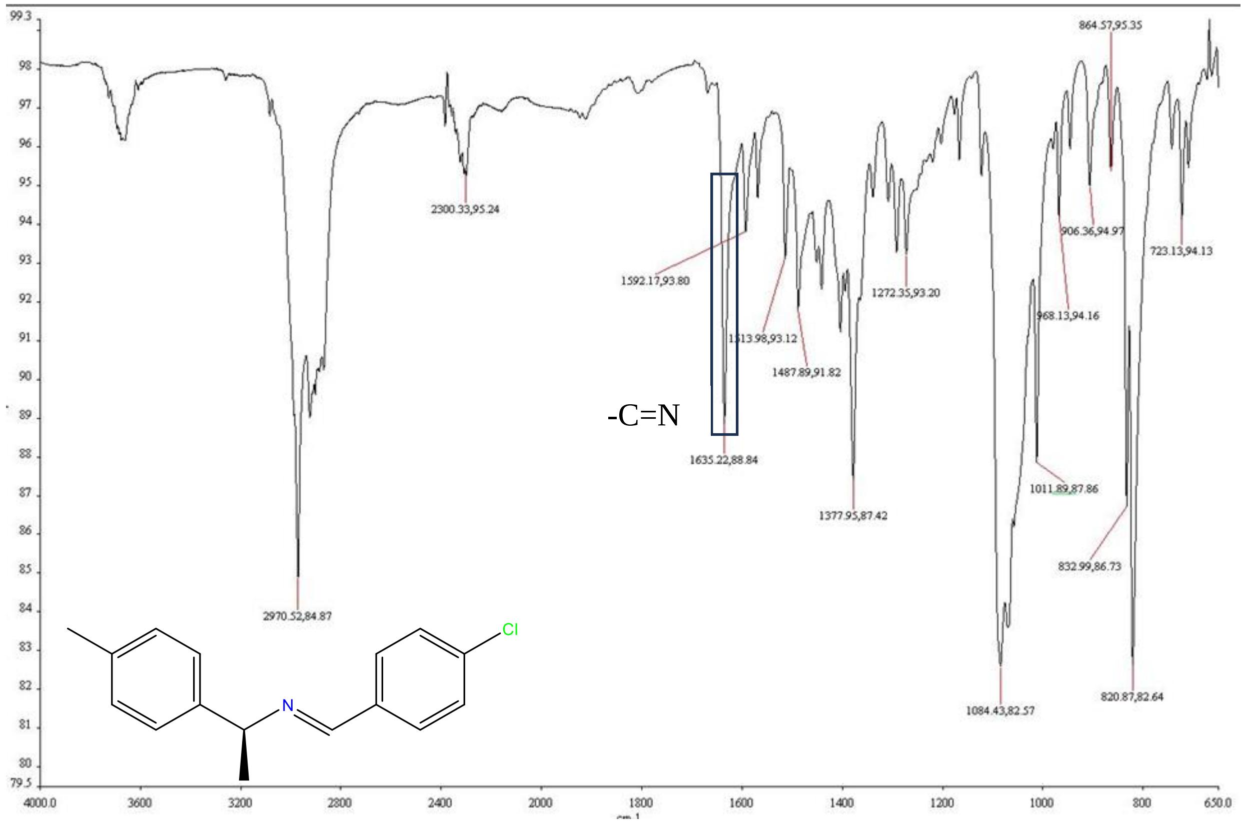

Figure S6. FT-IR spectrum of imine **II**

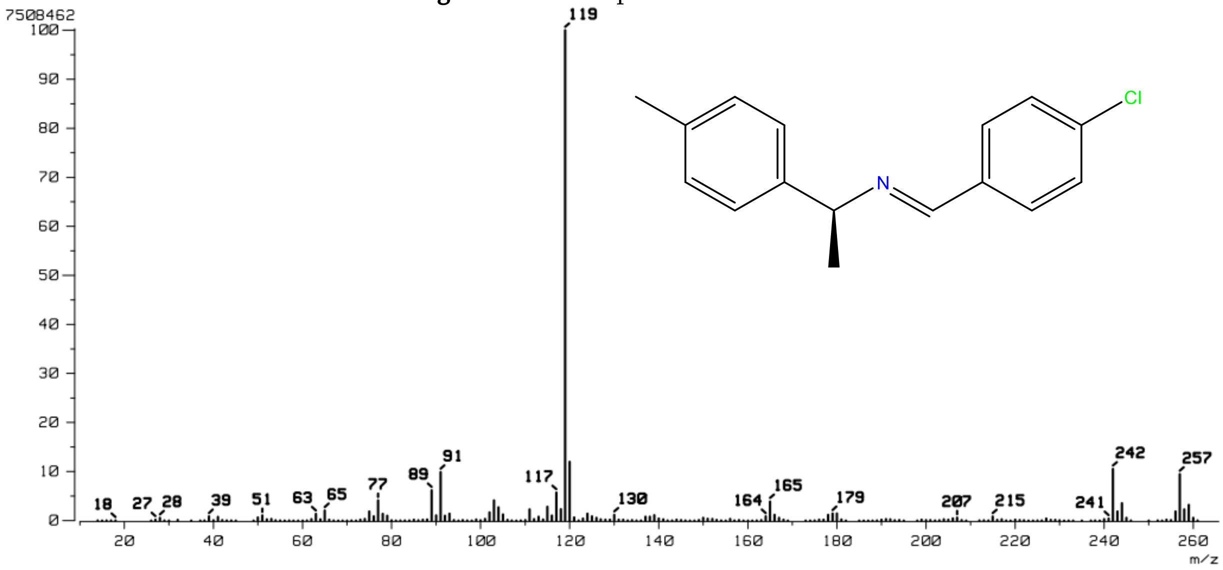

Figure S7. EI^+ mass spectrum of imine **II**

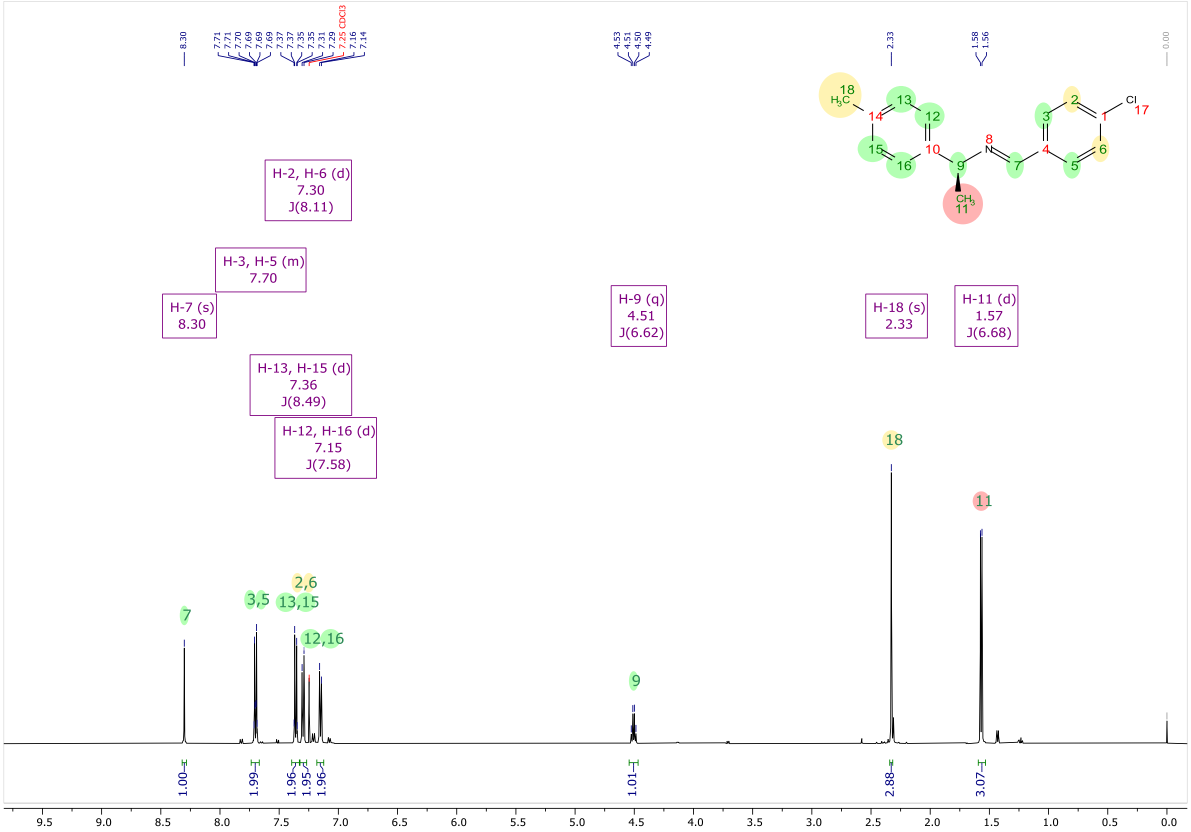

Figure S8. ^{1}H-NMR(500 MHz, $CDCl_3$) spectrum of imine **II**

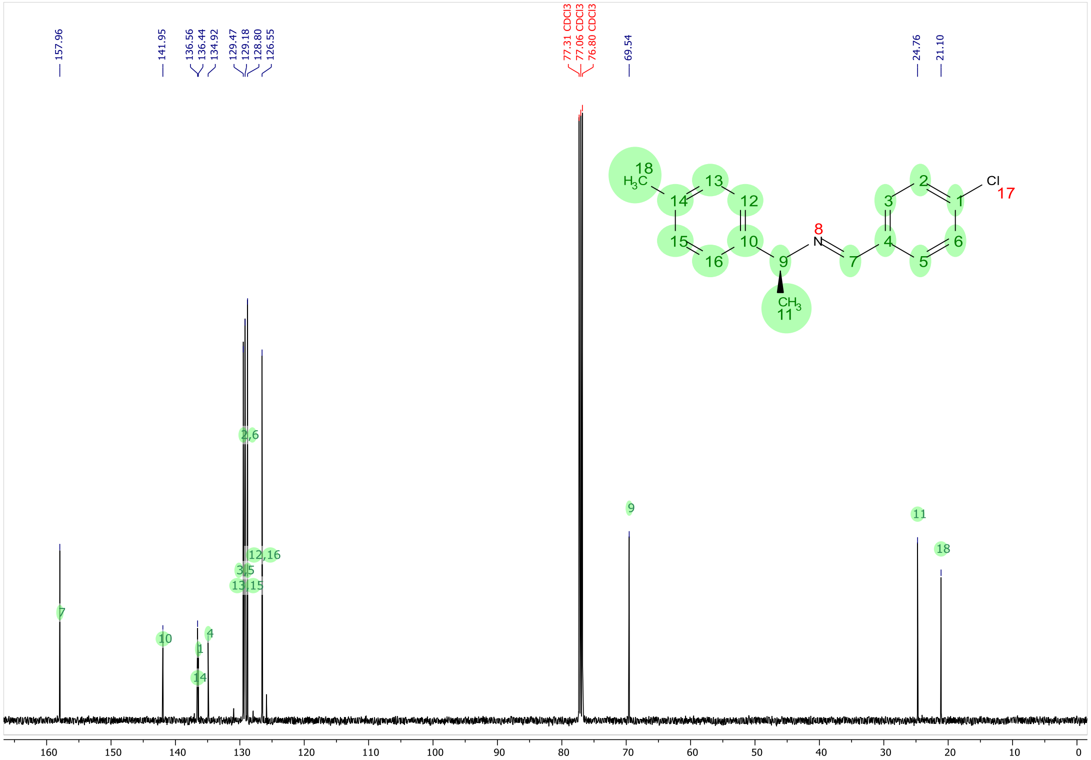

Figure S9. ^{13}C-NMR(125.5 MHz, $CDCl_3$) spectrum of imine **II**

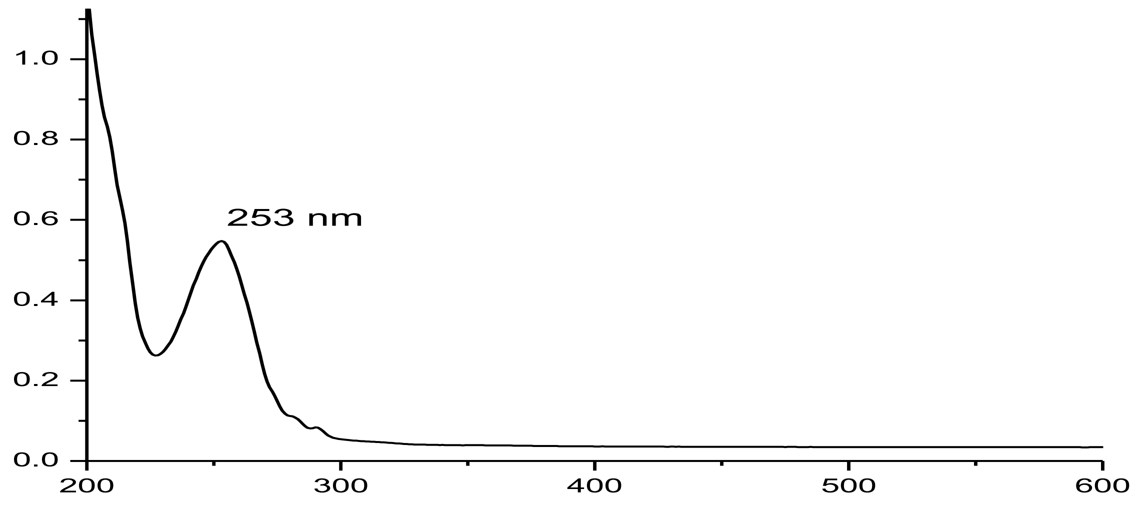

Figure S10. UV-Vis spectra (in acetonitrile) of imine **II**

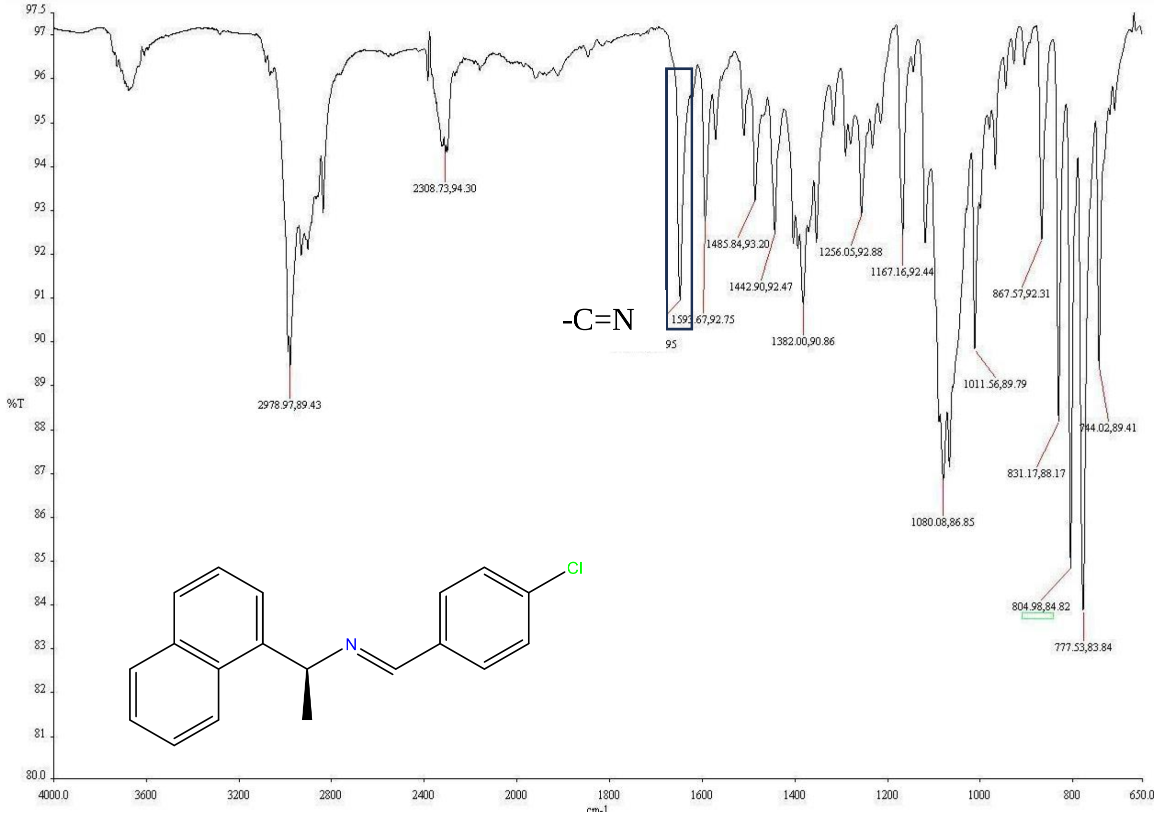

Figure S11. FT-IR spectrum of imine **III**

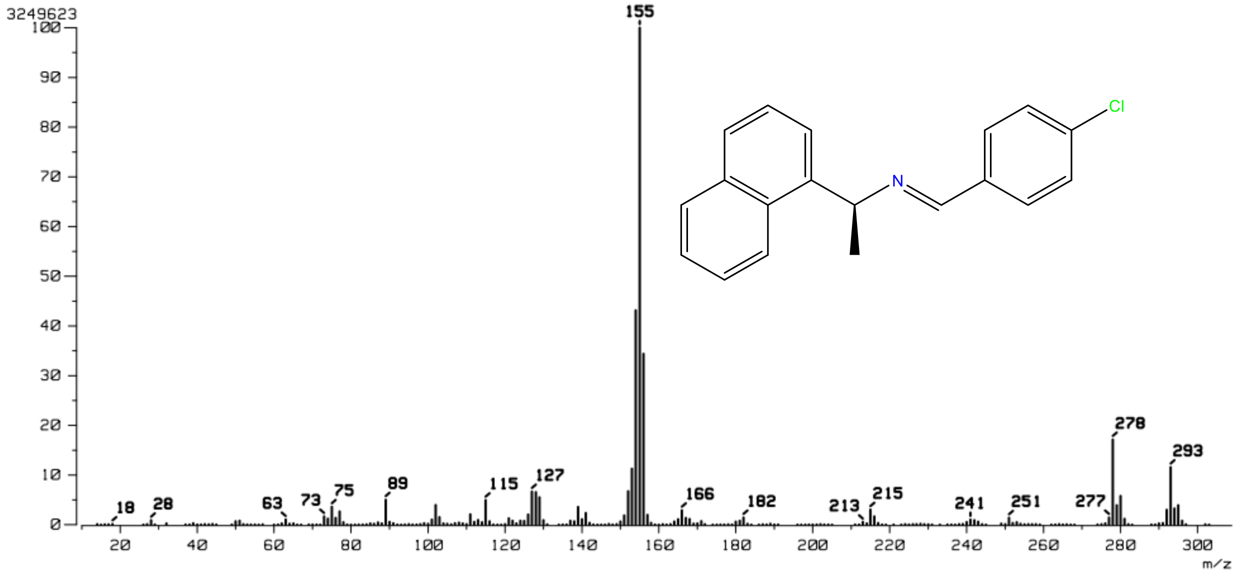

Figure S12. EI^+ mass spectrum of imine **III**

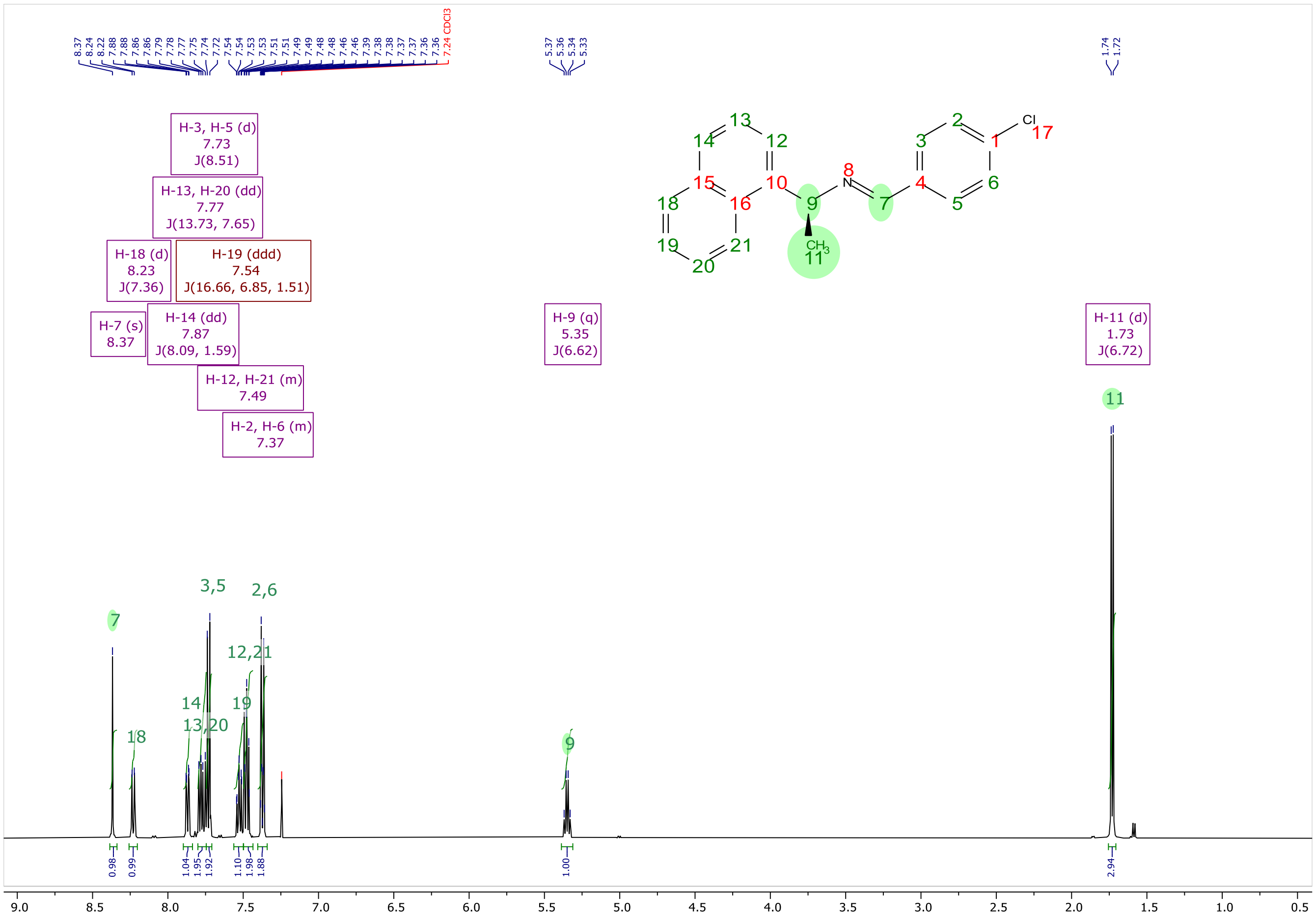

Figure S13. ^{1}H-NMR(500 MHz, $CDCl_3$) spectrum of imine **III**

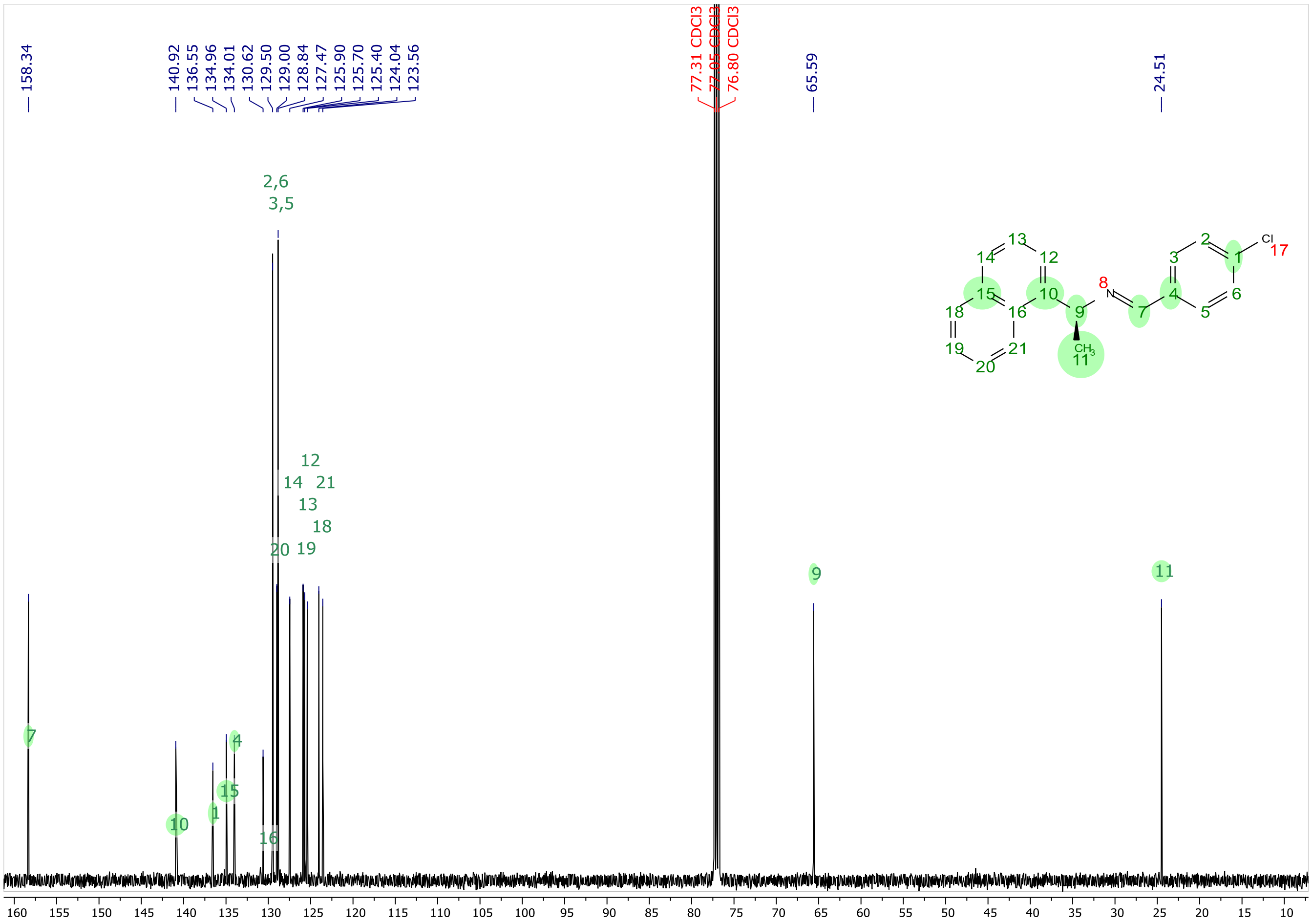

Figure S14. ^{13}C-NMR(125.5 MHz, $CDCl_3$) spectrum of imine **III**

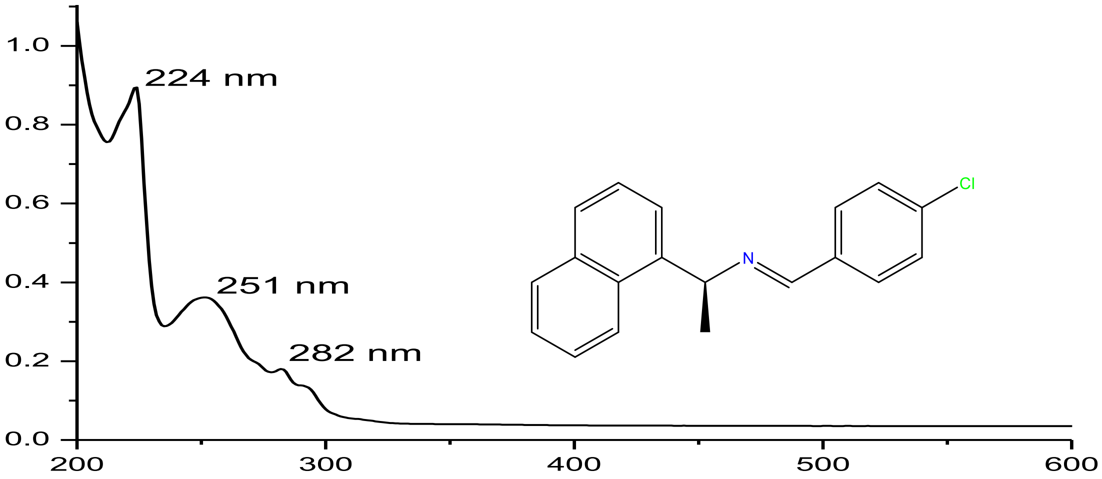

Figure S15. UV-Vis spectra (acetonitrile) of imine **III**

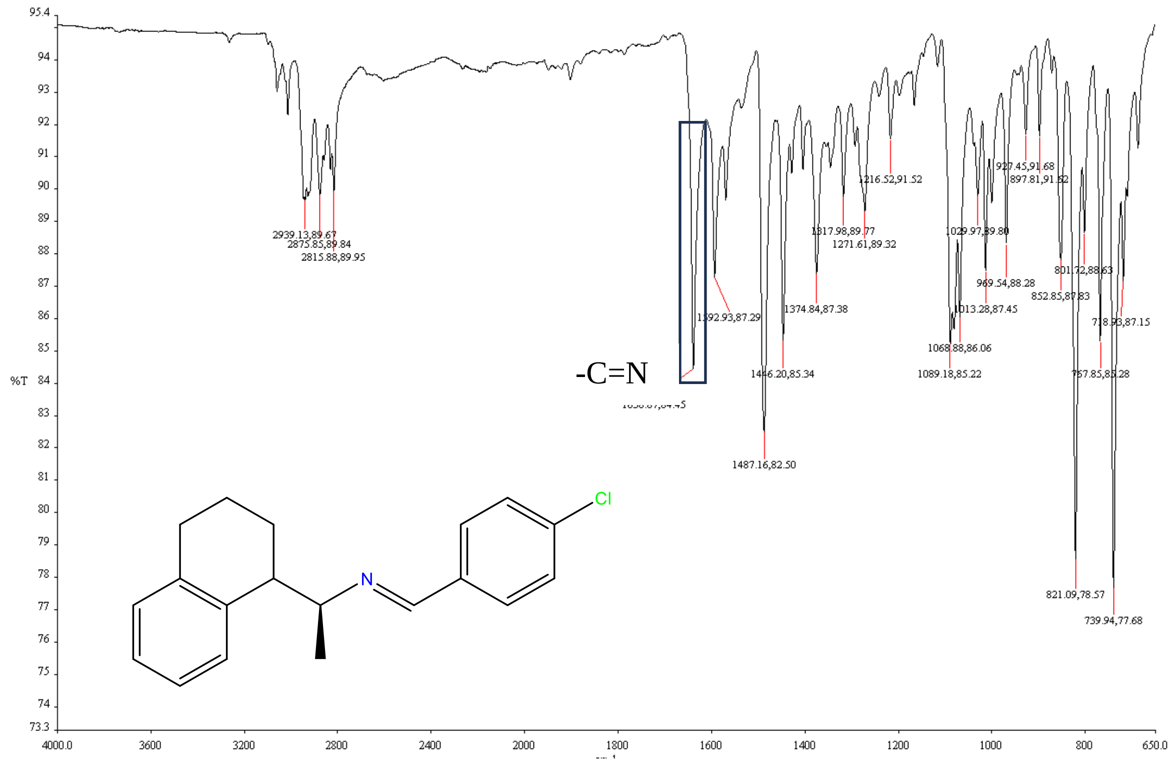

Figure S16. FT-IR spectrum of imine **IV**

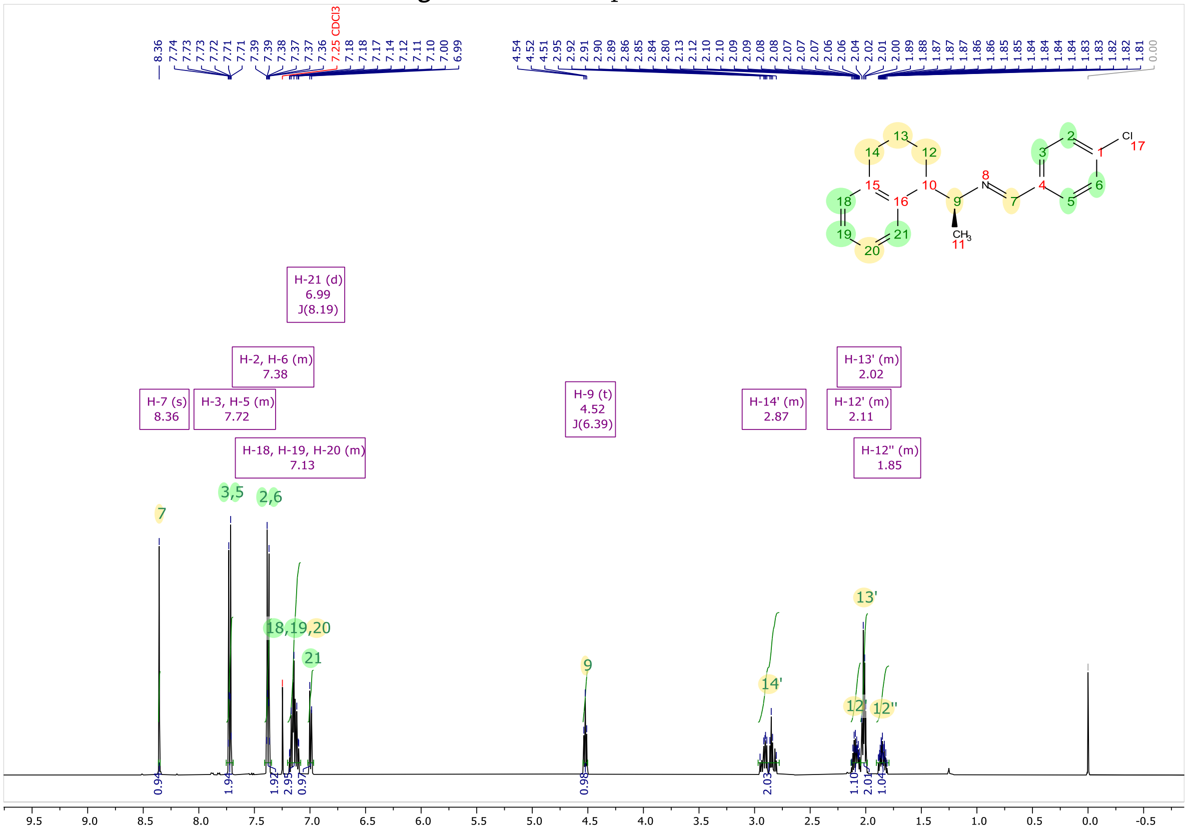

Figure S17. ^{1}H-NMR (500 MHz, CDCl3) spectrum of imine **IV**

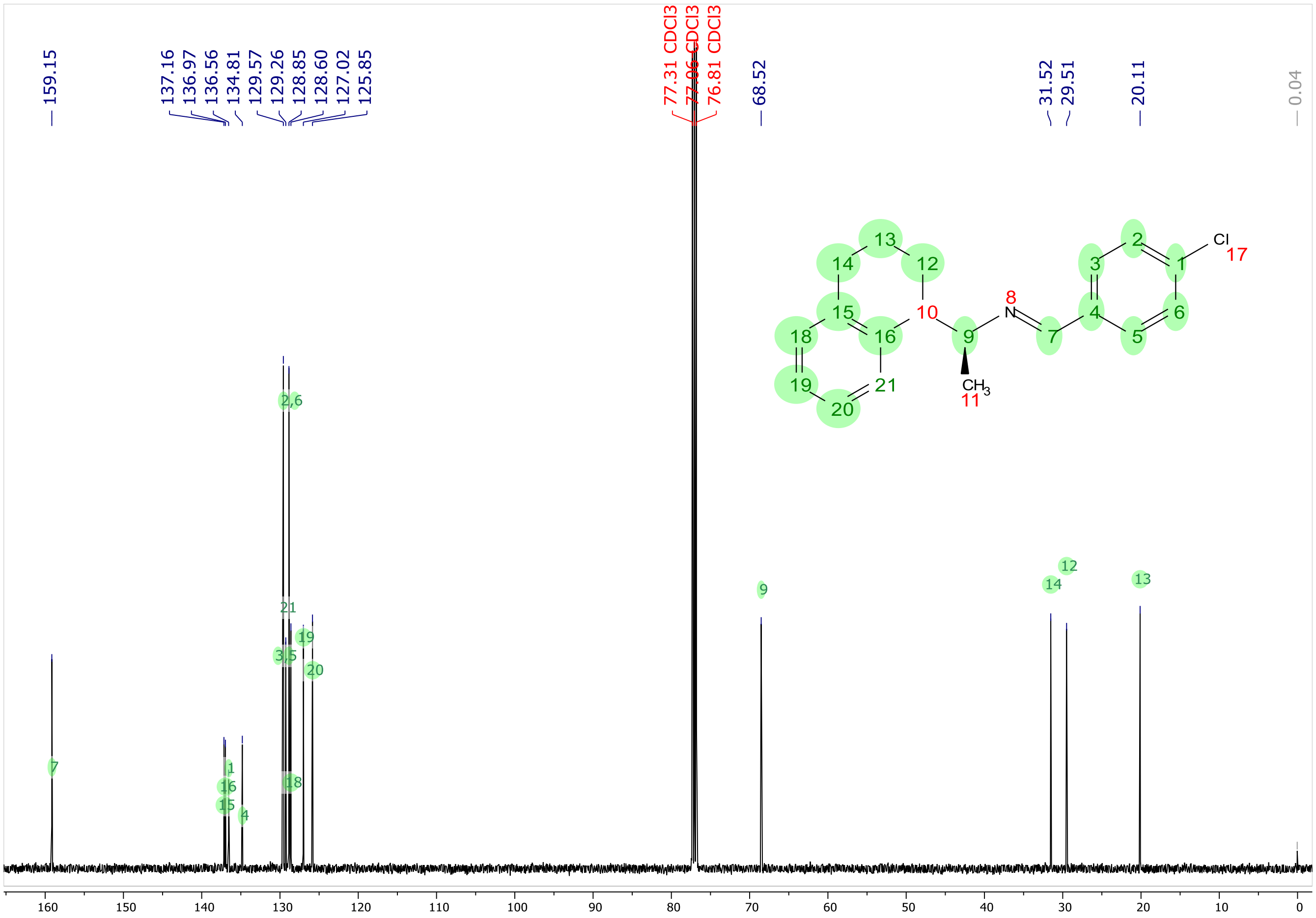

Figure S18. ^{13}C-NMR(125.5 MHz, $CDCl_3$) spectrum of imine **IV**

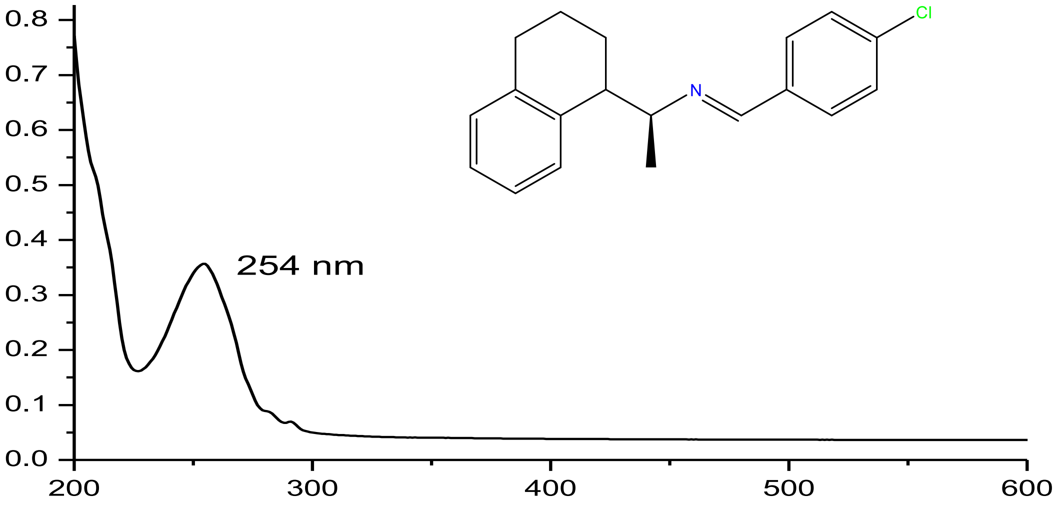

Figure S19. UV-Vis spectra (acetonitrile) of imine **IV**

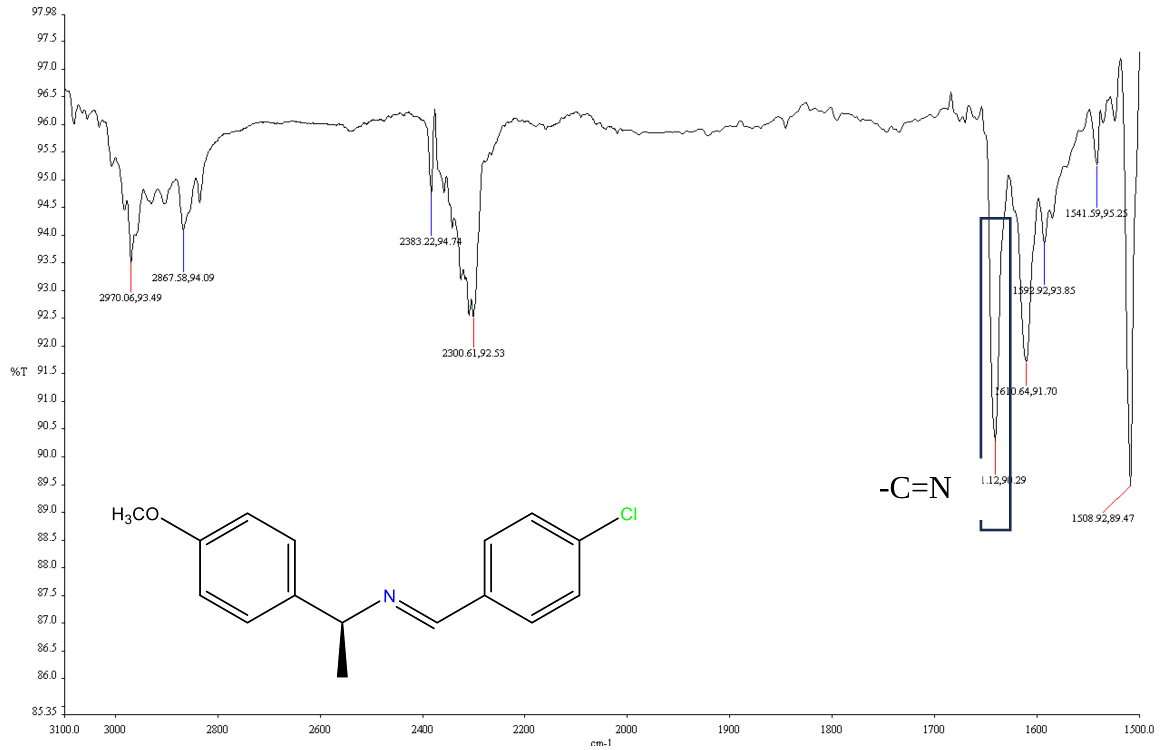

Figure S20. FT-IR spectrum of imine **V**

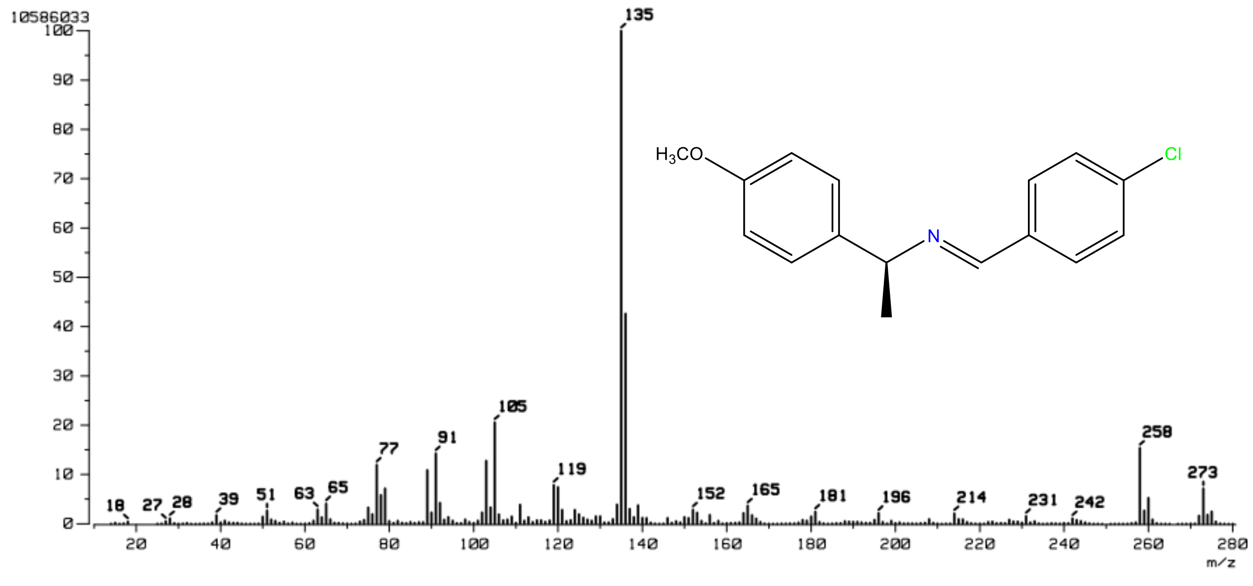

Figure S21. EI^+ mass spectrum of imine **V**

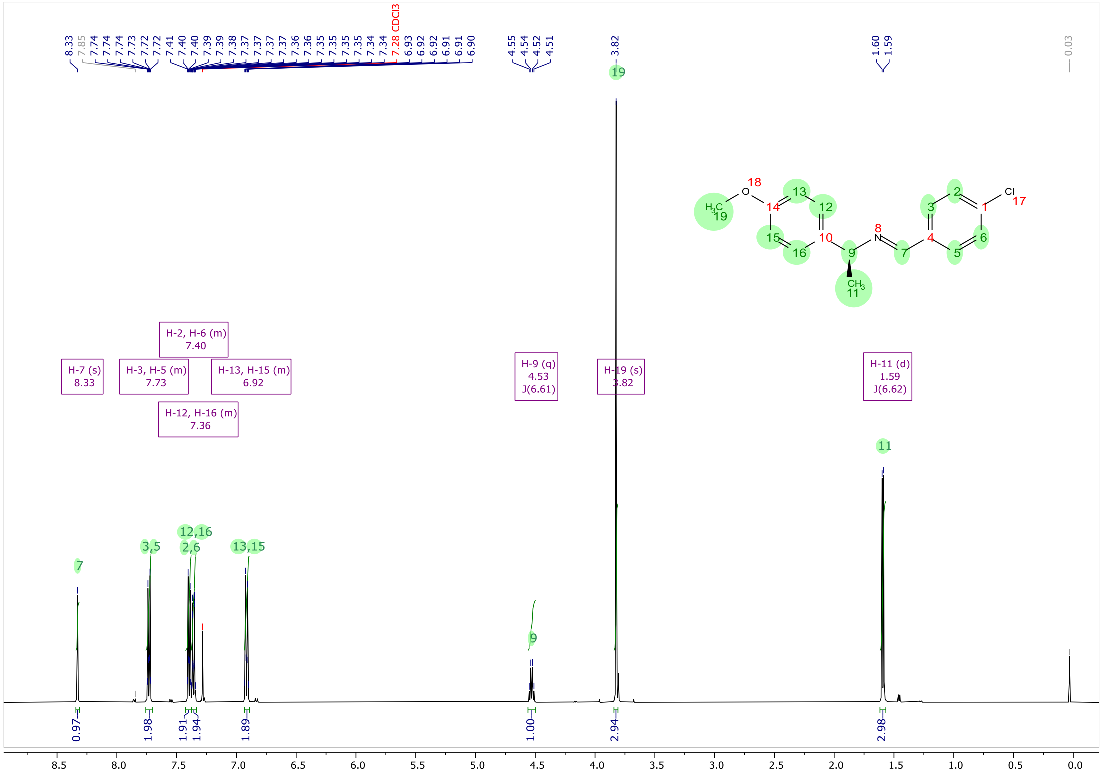

Figure S22. ^{1}H-NMR(500 MHz, $CDCl_3$) spectrum of imine **V**

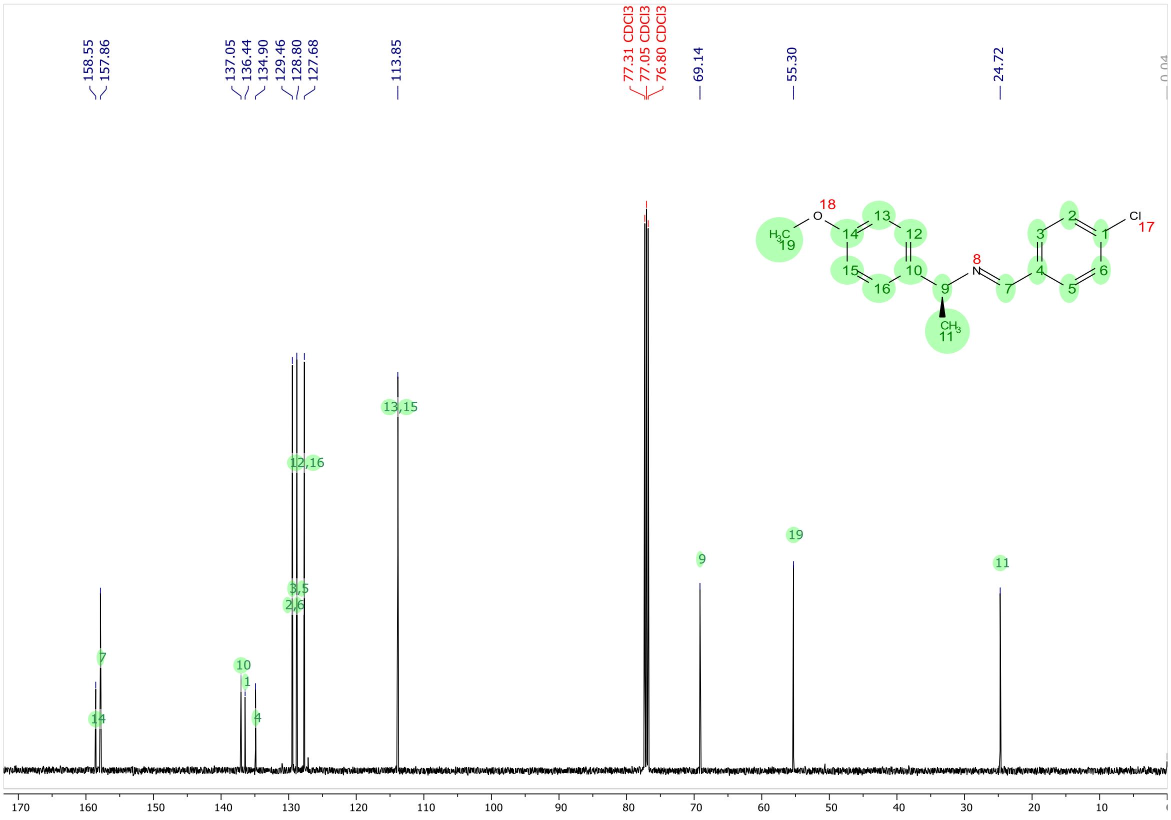

Figure S23. ^{13}C-NMR(125.5 MHz, $CDCl_3$) spectrum of imine **V**

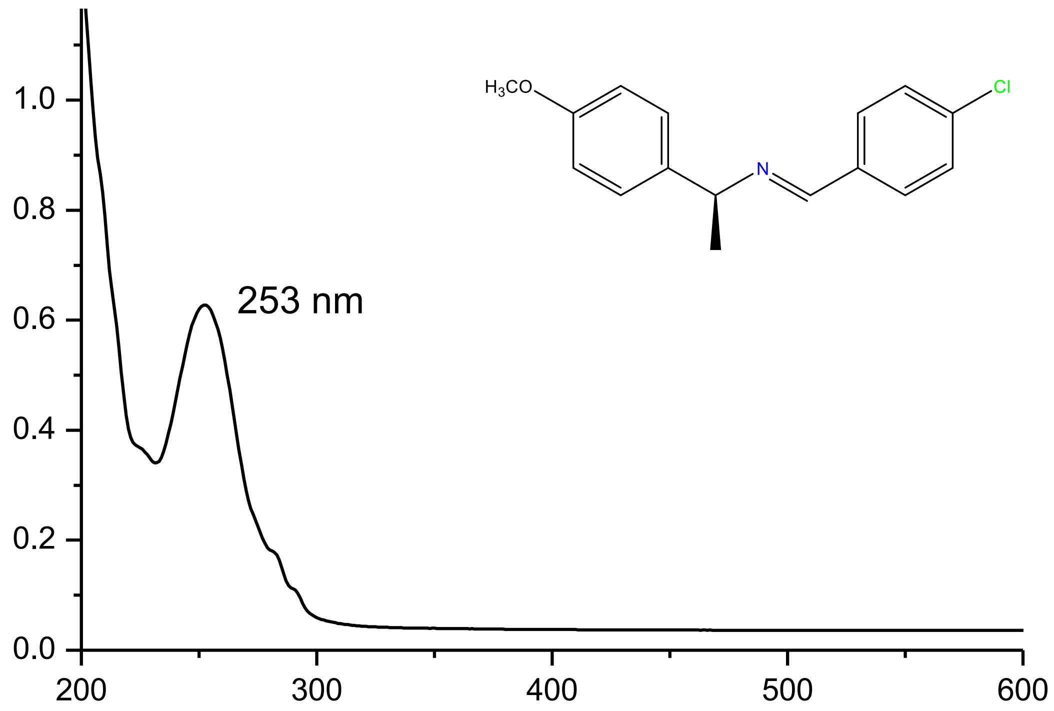

Figure S24. UV-Vis spectra (acetonitrile) of imine **V**

2. Crystallography data

Table S1. Experimental (X-ray) and theoretical (DFT) bond lengths and bond angles of chiral imine **I**, calculated at the B3LYP/6-311+G(d,p) and M06-2X/6-311+G(d,p) levels of theory.

IMINA I							
**Atoms**	**Bond lengths (Å)**			**Atoms**	**Bond angles (°)**		
	X-Ray	B3LYP	M06-2X		X-Ray	B3LYP	M06-2X
Cl(1)-C(8)	1.731(5)	1.75728	1.74312	C(3)-C(1)-C(9)	120.9(4)	119.50310	119.52726
C(1)-C(3)	1.381(7)	1.39929	1.39360	C(3)-C(1)-C(13)	118.1(4)	118.81652	119.29077
C(1)-C(9)	1.471(7))	1.47311	1.47615	C(13)-C(1)-C(9)	121.1(4)	121.68039	121.18192
C(1)-C(13)	1.394(6)	1.40249	1.39741	C(1)-C(3)-C(12)	121.6(4)	121.10088	120.87492
C(3)-C(12)	1.387(7)	1.39250	1.39023	C(9)-N(6)-C(2)	117.5(5)	118.29605	117.96913
N(6)-C(9)	1.255(7)	1.26916	1.26370	C(12)-C(8)-C(18)	121.0(5)	121.25350	121.41729
N(6)-C(2)	1.478(7)	1.46043	1.45579	N(6)-C(9)-C(1)	122.4(5)	123.30137	122.60831
C(8)-C(12)	1.376(7)	1.39017	1.38690	C(8)-C(12)-C(3)	119.0(4)	118.84726	118.75860
C(8)-C(18)	1.381(7)	1.39533	1.39234	C(18)-C(13)-C(1)	121.2(4)	120.72459	120.47342
C(13)-C(18)	1.383(7	1.38757	1.38507	C(4)-C(16)-C(2)	121.2(5)	119.99333	119.95829
C(16)-C(2)	1.500(8)	1.52346	1.51676	C(5)-C(16)-C(2)	120.9(5)	121.24053	120.92233
C(4)-C(11)	1.395(9)	1.39475	1.39160	C(5)-C(16)-C(4)	117.9(6)	118.74226	119.05966
C(7)-C(10)	1.324(11)	1.39492	1.39200	C(8)-C(18)-C(13)	119.2(4)	119.25724	119.18486
C(16)-C(5)	1.363(8)	1.39904	1.39494	C(16)-C(4)-C(11)	120.5(6)	120.82532	120.65041
C(7)-C(11)	1.373(11)	1.39297	1.39032	C(7)-C(11)C(4)	120.4(6)	120.04031	120.01627

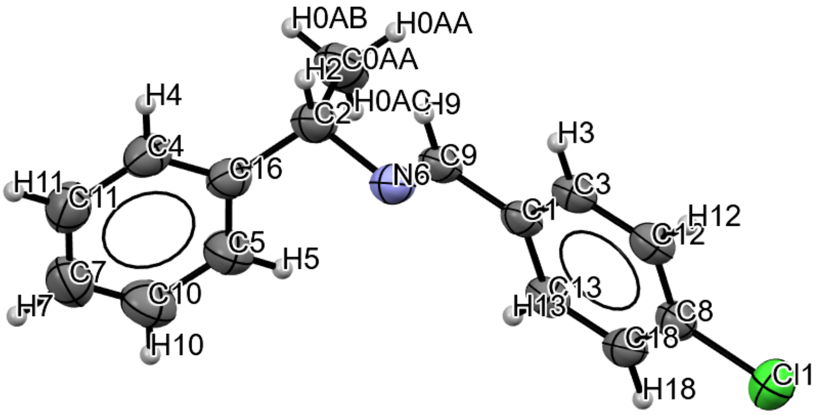

Figure S25

Table S2. Experimental (X-ray) and theoretical (DFT) bond lengths and bond angles of chiral imine **II**, calculated at the B3LYP/6-311+G(d,p) and M06-2X/6-311+G(d,p) levels of theory.

IMINA II							
**Atoms**	**Distances (Å)**			**Atoms**	**Angles (°)**		
	X-Ray	B3LYP	M06-2X		X-Ray	B3LYP	M06-2X
Cl(1)-C(1)	1.736(6)	1.75743	1.74253	C(7)-N(1)-C(8)	116.8(6)	118.27742	117.92289
N(1)-C(7)	1.254(8)	1.26915	1.26377	C(2)-C(1)-Cl(1)	119.2(6)	119.32747	119.27465
N(1)-C(8)	1.465(8)	1.46156	1.45727	C(6)-C(1)-Cl(1)	120.0(6)	119.42666	119.30589
C(1)-C(2)	1.370(9)	1.39527	1.39229	C(6)-C(1)-C(2)	120.7(6)	121.24587	121.41945
C(1)-C(6)	1.358(8)	1.39015	1.38709	C(1)-C(2)-C(3)	120.2(6)	119.26007	119.16829
C(2)-C(3)	1.398(9)	1.38764	1.38515	C(4)-C(3)-C(2)	119.8(6)	120.72776	120.48847
C(3)-C(4)	1.384(8)	1.40247	1.39754	C(3)-C(4)-C(7)	120.4(7)	121.69546	121.12620
C(4)-C(5)	1.379(8)	1.39923	1.39362	C(5)-C(4)-C(3)	118.6(6)	118.80877	119.29234
C(4)-C(7)	1.472(9)	1.47319	1.47610	C(4)-C(5)-C(6)	121.3(6)	121.10554	120.85811
C(5)-C(6)	1.399(8)	1.39249	1.39026	C(1)-C(6)-C(5)	119.2(7)	118.85197	118.77316
C(8)-C(9)	1.539(10)	1.53892	1.53138	N(1)-C(7)-C(4)	122.4(7)	123.31944	122.62847
C(8)-C(10)	1.519(10)	1.52247	1.51624	N(1)-C(8)-C(10)	110.4(6)	110.13736	109.91457
C(10)-C(15)	1.377(9)	1.39437	1.39169	C(11)-C(10)-C(15)	118.1(8)	118.18708	118.59750
C(11)-C(12)	1.395(10)	1.39031	1.38868	C(13)-C(12)-C(11)	119.1(9)	121.32736	121.20699

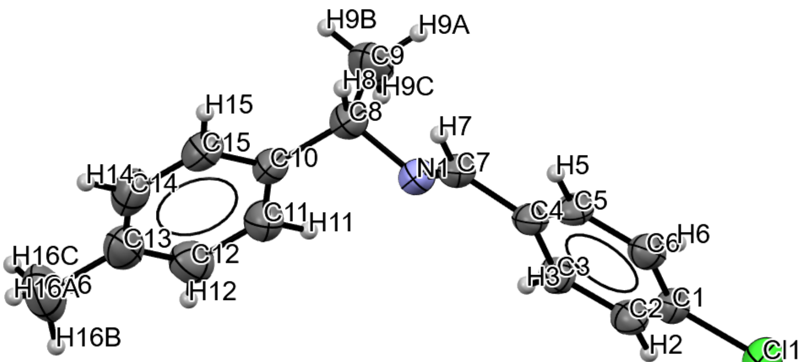

Figure S26

Table S3. Experimental (X-ray) and theoretical (DFT) bond lengths and bond angles of chiral imine **III**, calculated at the B3LYP/6-311+G(d,p) and M06-2X/6-311+G(d,p) levels of theory.

IMINA III							
**Atoms**	**Distances (Å)**			**Atoms**	**Angles (°)**		
	X-Ray	B3LYP	M06-2X		X-Ray	B3LYP	M06-2X
Cl(21)-C(18)	1.738(3)	1.75699	1.74270	C(8)-C(7)-C(2)	119.6(2)	119.57810	119.62641
C(7)-C(2)	1.423(3)	1.43508	1.42400	C(8)-C(7)-C(6)	121.3(3)	121.01963	120.86140
C(7)-C(8)	1.408(4)	1.41857	1.41872	C(7)-C(2)-C(1)	118.8(2)	118.88739	118.96181
C(7)-C(6)	1.422(3)	1.41989	1.41874	C(20)-C(15)-C(13)	120.4(2)	119.50143	119.51075
C(2)-C(1)	1.430(3)	1.43462	1.43231	N(12)-C(11)-C(1)	110.7(2)	110.92912	110.84210
C(2)-C(3)	1.416(3)	1.42333	1.42254	C(4)-C(3)-C(2)	121.2(3)	121.51566	121.26502
C(1)-C(10)	1.373(3)	1.37882	1.37260	C(19)-C(18)-Cl(21)	119.8(2)	119.42435	119.37413
C(1)-C(11)	1.509(3)	1.52770	1.52005	C(19)-C(18)-C(17)	121.4(3)	121.25288	121.39512
C(10)-C(9)	1.405(4)	1.41254	1.41389	C(17)-C(18)-Cl(21)	118.8(3)	119.32274	119.23068
C(15)-C(13)	1.468(3)	1.47306	1.47588	C(16)-C(17)-C(18)	118.9(3)	119.24995	119.19613
C(15)-C(20)	1.385(3)	1.39930	1.39370	C(6)-C(5)-C(4)	120.3(3)	119.70450	119.72588
N(12)-C(11)	1.462(3)	1.46043	1.45577	C(18)-C(19)-C(20)	119.4(2)	118.85694	118.77215
N(12)-C(13)	1.255(3)	1.26921	1.26381	C(1)-C(11)-C(14)	110.4(2)	111.12484	110.44684

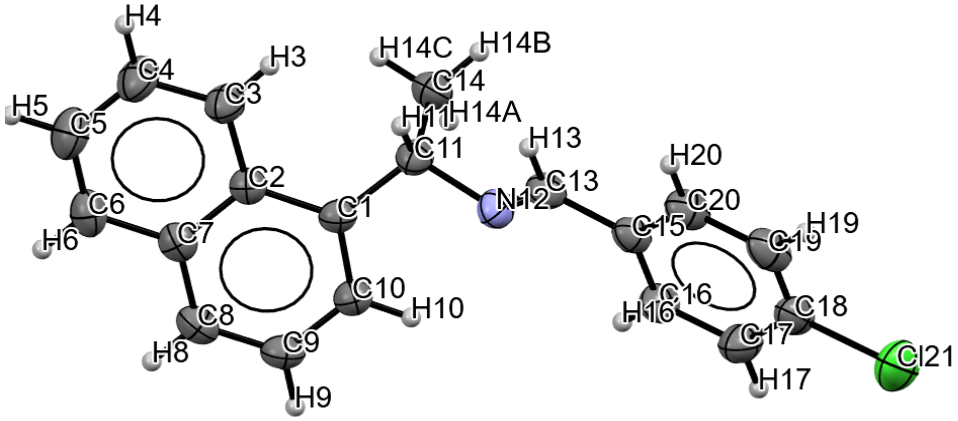

Figure S27

Table S4. Experimental (X-ray) and theoretical (DFT) bond lengths and bond angles of chiral imine **IV**, calculated at the B3LYP/6-311+G(d,p) and M06-2X/6-311+G(d,p) levels of theory.

IMINA IV							
**Atoms**	**Distances (Å)**			**Atoms**	**Angles (°)**		
	X-Ray	B3LYP	M06-2X		X-Ray	B3LYP	M06-2X
C(15)-Cl(1)	1.741(2)	1.75732	1.74227	C(11)-N(1)-C(1)	117.94(19)	118.37790	117.96664
N(1)-C(1)	1.464(3)	1.45806	1.45436	C(4)-C(3)-C(2)	121.6(2)	121.37859	121.21244
C(11)-C(12)	1.470(3)	1.47303	1.47592	C(5)-C(4)-C(3)	119.3(3)	119.56828	119.55750
C(3)-C(2)	1.395(3)	1.40052	1.39697	C(6)-C(5)-C(4)	119.9(3)	119.47422	119.53920
N(1)-C(11)	1.255(3)	1.27022	1.26477	C(5)-C(6)-C(7)	121.6(2)	121.53642	121.43655
C(7)-C(8)	1.512(4)	1.51744	1.51414	C(2)-C(7)-C(6)	118.8(2)	118.91354	118.90789
C(1)-C(10)	1.533(3)	1.53691	1.52886	C(9)-C(8)-C(7)	113.1(2)	113.06782	112.61077
C(16)-C(17)	1.381(3)	1.39245	1.39017	C(8)-C(9)-C(10)	109.7(2)	112.61077	109.68167
C(13)-C(14)	1.381(4)	1.38768	1.38522	N(1)-C(1)-C(10)	108.24(19)	108.87961	108.94073
C(8)-C(9)	1.504(4)	1.53024	1.52554	C(3)-C(2)-C(1)	119.1(2)	119.32465	119.13028
C(9)-C(10)	1.510(4)	1.52889	1.52503	C(17)-C(12)-C(11)	121.80(18)	119.53997	119.55570
C(4)-C(5)	1.378(4)	1.39426	1.39213	C(14)-C(15)-Cl(1)	119.45(17)	119.32715	119.24781
C(5)-C(6)	1.372(5)	1.38976	1.38682	C(2)-C(1)-C(10)	111.88(19)	112.20140	111.86674
C(6)-C(7)	1.394(4)	1.40086	1.39737	C(16)-C(15)-Cl(1)	119.21(19)	119.40790	119.32947
C(7)-C(2)	1.392(3)	1.40501	1.39993	C(16)-C(17)-C(12)	120.78(19)	121.08807	120.85528

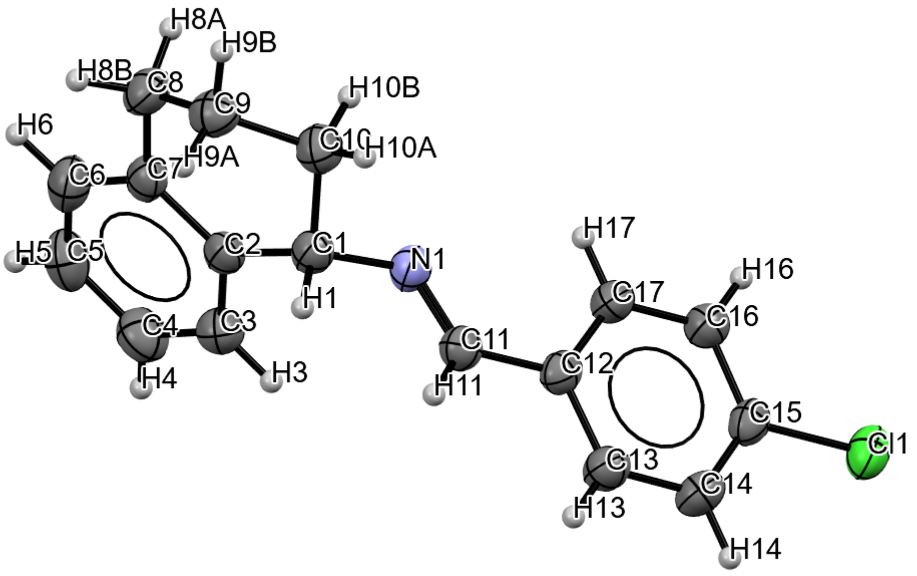

Figure S28

Table S5. Experimental (X-ray) and theoretical (DFT) bond lengths and bond angles of chiral imine **V**, calculated at the B3LYP/6-311+G(d,p) and M06-2X/6-311+G(d,p) levels of theory.

IMINA V							
**Atoms**	**Distances (Å)**			**Atoms**	**Angles (°)**		
	X-Ray	B3LYP	M06-2X		X-Ray	B3LYP	M06-2X
Cl(1)-C(14)	1.743(4)	1.75754	1.74230	C(2)-N(1)-C(1)	117.5(4)	118.35660	117.78753
N(1)-C(2)	1.260(5)	1.26908	1.26379	C(12)-C(11)-C(2)	120.3(4)	119.52920	119.45473
O(1)-C(7)	1.371(5)	1.36645	1.35943	C(15)-C(14)-Cl(1)	118.5(3)	119.32587	119.26159
O(1)-C(10)	1.417(6)	1.42019	1.41090	C(13)-C(14)-C(15)	121.3(4)	121.25269	121.40936
C(11)-C(12)	1.389(5)	1.39927	1.39360	N(1)-C(2)-C(11)	122.3(4)	123.27854	122.77251
C(11)-C(16)	1.396(6)	1.40249	1.39744	C(9)-C(4)-C(5)	116.9(4)	118.00114	118.35937
C(11)-C(2)	1.473(6)	1.47329	1.47592	N(1)-C(1)-C(4)	109.8(3)	110.00093	109.89973
C(15)-C(16)	1.364(6)	1.38766	1.38529	N(1)-C(1)-C(3)	107.2(4)	108.71402	108.49865
C(15)-C(14)	1.383(5)	1.39526	1.39242	O(1)-C(7)-C(8)	124.8(4)	124.69910	124.53554
C(12)-C(13)	1.375(6)	1.39253	1.39022	C(5)-C(6)-C(7)	120.0(4)	120.26717	120.22728
C(14)-C(13)	1.370(5)	1.39015	1.38705	C(6)-C(5)-C(4)	122.4(5)	121.09053	120.82931
C(4)-C(1)	1.506(6)	1.52202	1.51524	C(7)-C(8)-C(9)	119.9(5)	119.44833	119.24977
C(4)-C(9)	1.372(6)	1.39165	1.38650	C(9)-C(4)-C(1)	121.3(4)	120.62503	120.58285
C(4)-C(5)	1.381(6)	1.40274	1.39985				
C(1)-C(3)	1.532(6)	1.53863	1.53115				
C(7)-C(8)	1.373(6)	1.39506	1.39140				
C(9)-C(8)	1.395(7)	1.39858	1.39653				

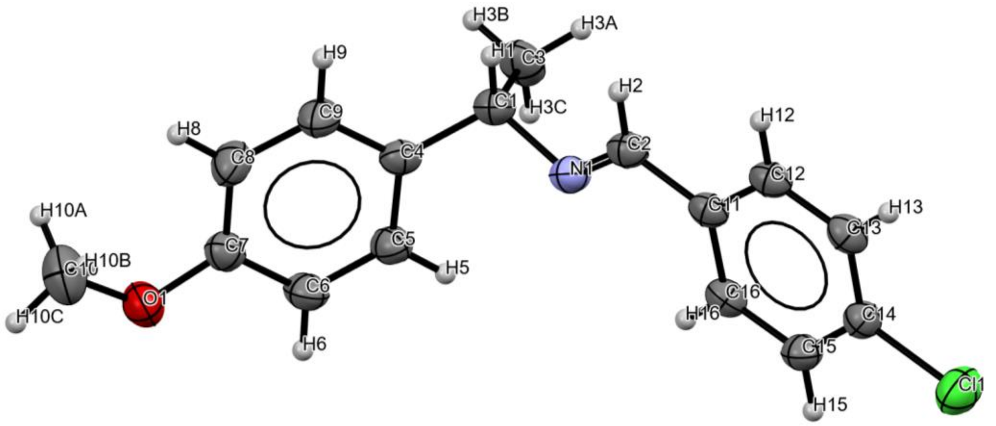

Figure S29

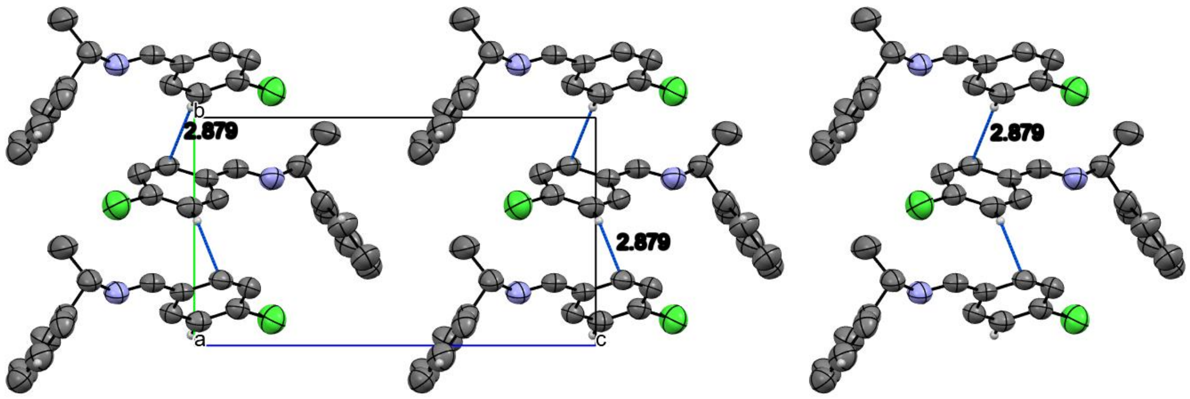

Imine I

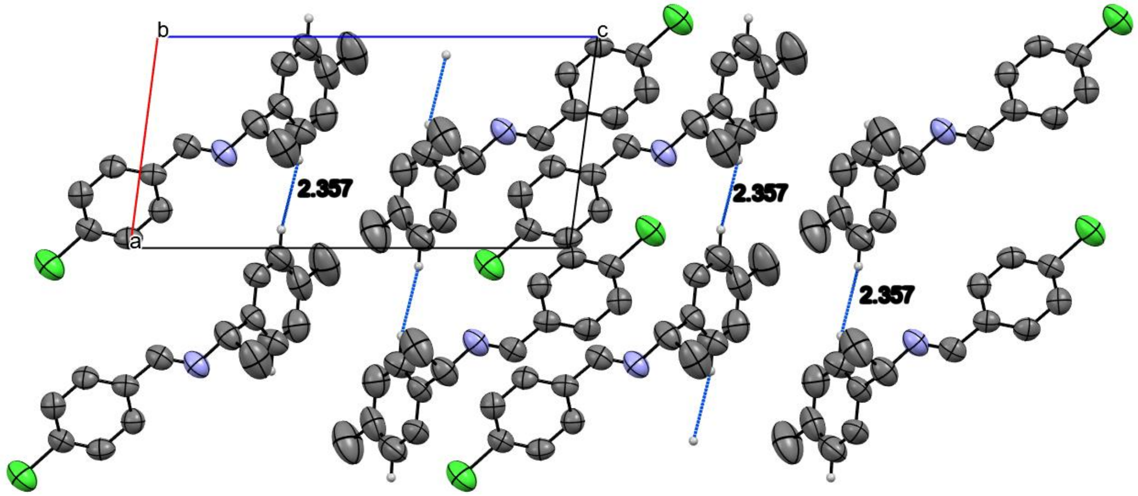

Imine II

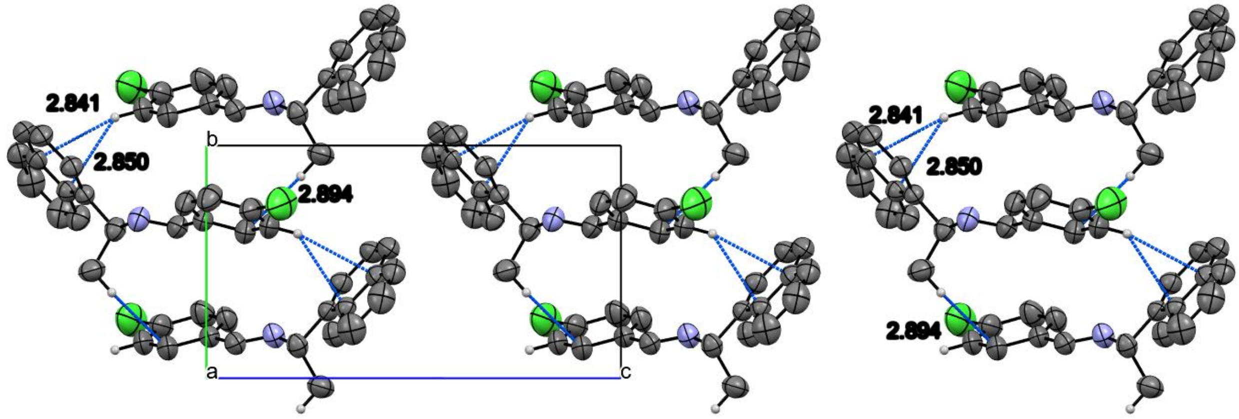

Imine III

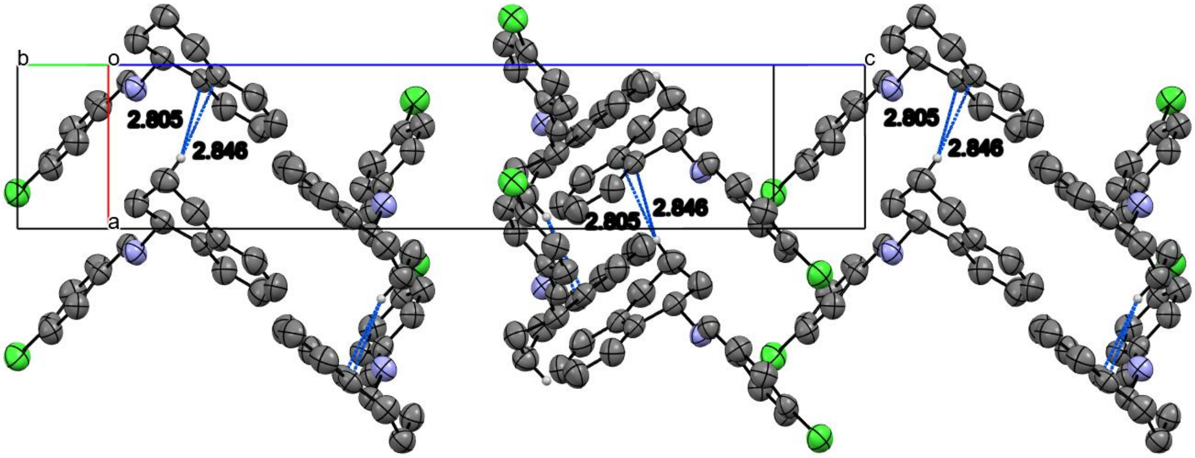

Imine IV

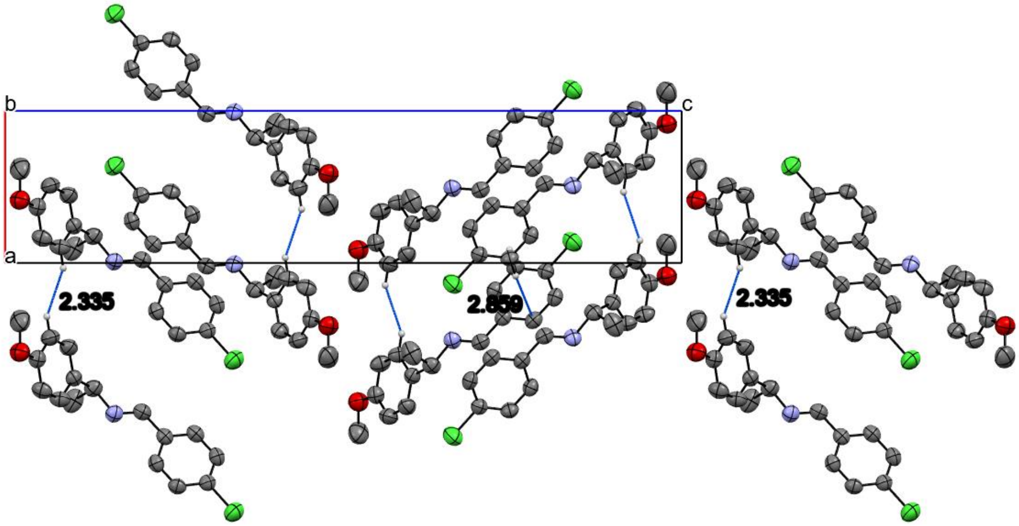

Imine V

Figure S30. The crystal packing diagrams of compounds **I–V** are observed along the b axis. The dashed blue lines indicate intermolecular contact. For clarity, all H atoms not involved in these interactions have been omitted. Displacement ellipsoids are at the 40% probability level.

Table S6. Root mean squared error (RMSE), mean absolute error (MAE), and Pearson correlation coefficient (r) values for statistical comparison between experimental (X-ray) and theoretical bond lengths and bond angles calculated using B3LYP/6-311+G(d,p) and M06-2X/6-311+G(d,p) for imines **I–V**.

		IMINES											
		I		II		III		IV		V		AVERAGE	
		B3LYP	M062X	B3LYP	M062X	B3LYP	M062X	B3LYP	M062X	B3LYP	M062X	B3LYP	M062X
Bond Lengths (Å)	RMSE	0.0247	0.0220	0.0156	0.0131	0.0107	0.0069	0.0130	0.0099	0.0138	0.0110	***0.0156***	***0.0126***
	MAE	0.0180	0.0149	0.0126	0.0112	0.0092	0.0060	0.0112	0.0081	0.0120	0.0089	***0.0126***	***0.0098***
	r	0.9826	0.9832	0.9928	0.9937	0.9983	0.9987	0.9973	0.9974	0.9967	0.9960	***0.9935***	***0.9938***
Bond Angles (°)	RMSE	0.6954	0.7301	0.9087	0.8287	0.4649	0.4206	0.9900	0.6464	0.8254	0.8318	***0.7769***	***0.6915***
	MAE	0.5822	0.5581	0.7025	0.6884	0.3932	0.3161	0.5695	0.3490	0.7000	0.6680	***0.5895***	***0.5159***
	r	0.9610	0.9650	0.9577	0.9603	0.9931	0.9942	0.9794	0.9904	0.9880	0.9874	***0.9758***	***0.9795***

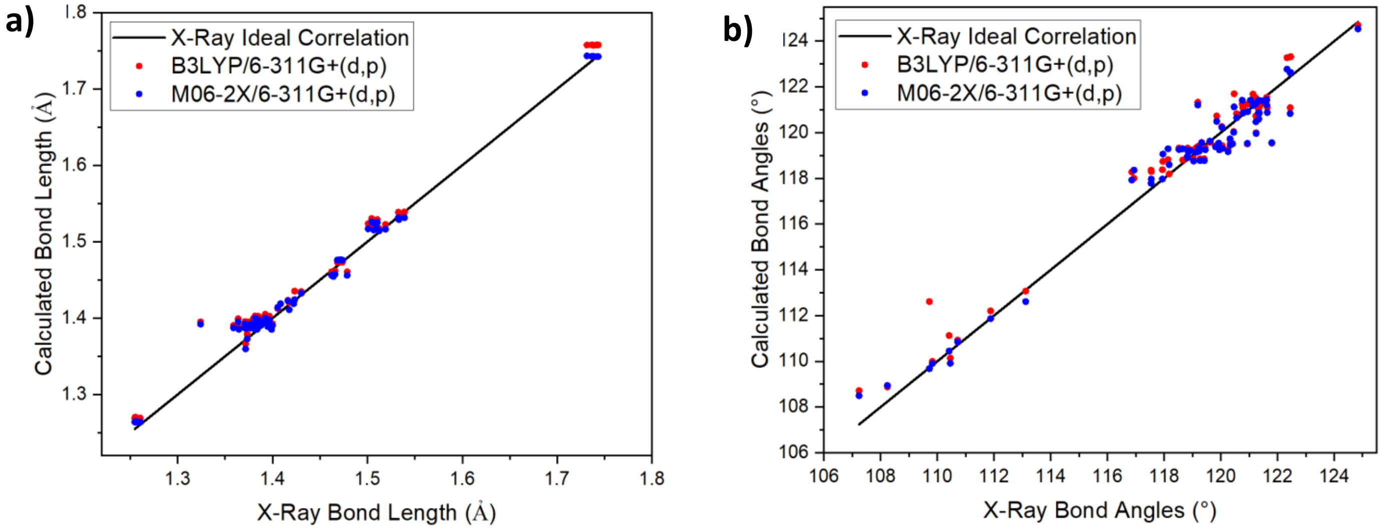

Figure S31. Correlation between experimental **a)** bond lenghts and **b)** bond angles obtained from single-crystal X-ray diffraction and theoretical values calculated using B3LYP/6-311+G(d,p) (red) and M06-2X/6-311+G(d,p) (blue) for imines **I - V**. The solid diagonal line represents perfect agreement.

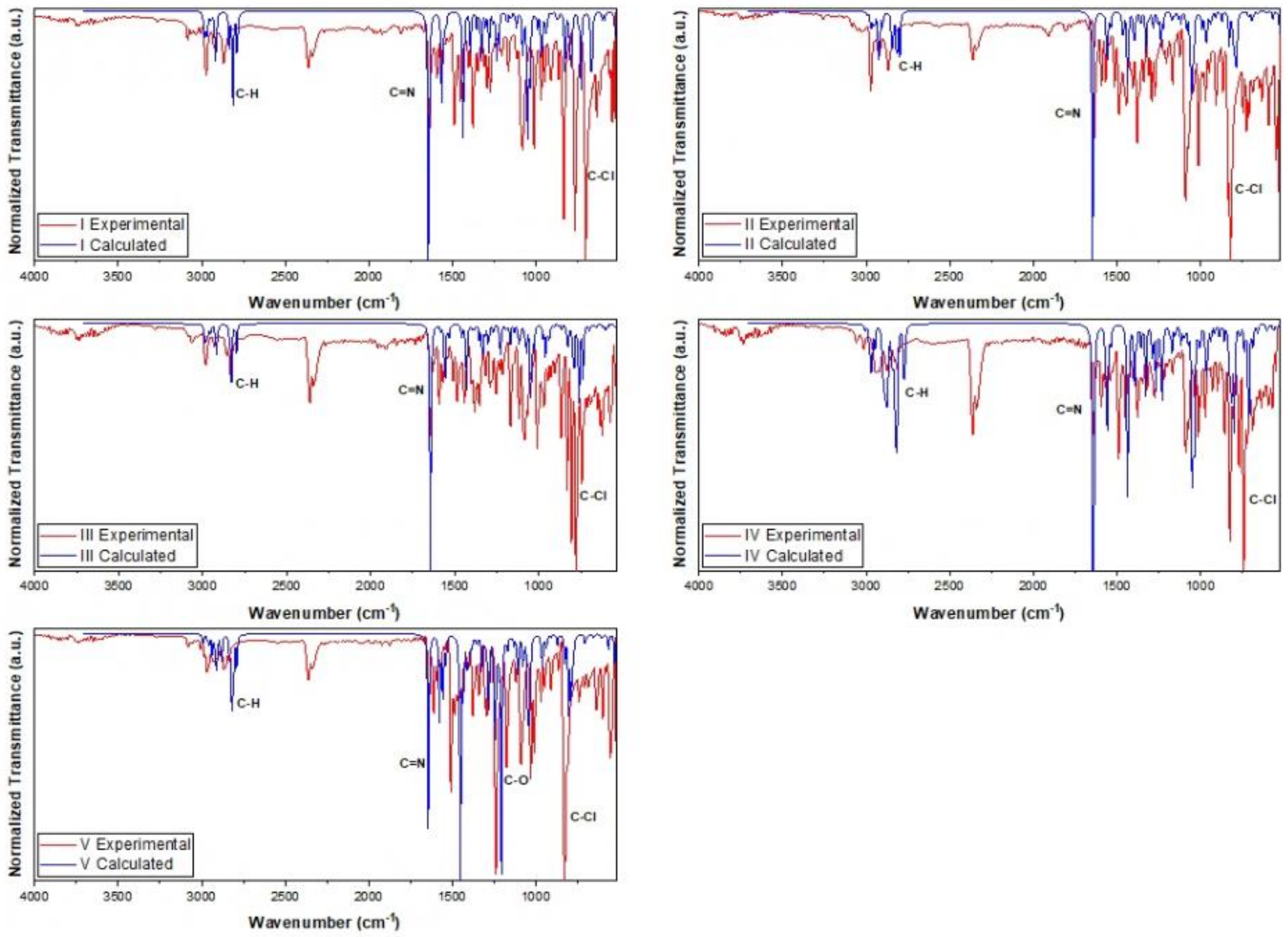

Figure S32. Comparison of experimental (red) and M06-2X/6-311+G(d,p) calculated (blue) IR spectra for the chiral imines **I-V**.

Table S7. NBO analysis for imine **I** at the M06-2X/6-311+G(d,p) level.

IMINE I								
Donor (i)	Type	ED/e	Acceptor	Type	ED/e	E(2) (kcal/mol)	E(j)-E(i) (a.u.)	F(i,j) (a.u.)
N2-C3	π	1.94776	C5-C13	π*	0.3618	9.19	0.44	0.062
C5-C13	π	1.64764	N2-C3	π*	0.08835	17.5	0.38	0.078
C5-C13	π	1.64764	C6-C8	π*	0.28563	27.24	0.36	0.09
C5-C13	π	1.64764	C10-C11	π*	0.37987	29.23	0.34	0.089
C6-C8	π	1.66666	C5-C13	π*	0.3618	26.94	0.36	0.088
C6-C8	π	1.66666	C10-C11	π*	0.37987	30.69	0.34	0.092
C10-C11	π	1.68017	C5-C13	π*	0.3618	27.13	0.38	0.091
C10-C11	π	1.68017	C6-C8	π*	0.28563	24.4	0.38	0.087
C17-C26	π	1.66161	C18-C20	π*	0.32097	27.22	0.36	0.089
C17-C26	π	1.66161	C22-C24	π*	0.33332	29.9	0.36	0.092
C18-C20	π	1.66313	C17-C26	π*	0.34515	30.57	0.36	0.094
C18-C20	π	1.66313	C22-C24	π*	0.33332	28.8	0.35	0.09
C22-C24	π	1.67003	C17-C26	π*	0.34515	27.54	0.36	0.089
C22-C24	π	1.67003	C18-C20	π*	0.32097	28.6	0.36	0.091
LP-Cl1		1.9293	C10-C11	π*	0.37987	15.68	0.42	0.079
C5-C13	π	1.6382	N2-C3	π*	0.08835	58.61	0.02	0.072
C10-C11	π	1.62013	C5-C13	π*	0.3618	250.61	0.02	0.1
C10-C11	π	1.62013	C6-C8	π*	0.28563	149.98	0.02	0.093

Table S8. NBO analysis for imine **II** at the M06-2X/6-311+G(d,p) level.

IMINE II								
**Donor (i)**	**Type**	**ED/e**	**Acceptor**	**Type**	**ED/e**	**E(2) (kcal/mol)**	**E(j)-E(i) (a.u.)**	**F(i,j) (a.u.)**
N2-C13	π	1.9473	C8-C9	π*	0.36153	9.25	0.44	0.062
C3-C11	π	1.68055	C4-C6	π*	0.28589	24.4	0.38	0.087
C3-C11	π	1.68055	C8-C9	π*	0.36153	27.07	0.38	0.091
C4-C6	π	1.66664	C3-C11	π*	0.38048	30.72	0.34	0.092
C4-C6	π	1.66664	C8-C9	π*	0.36153	26.92	0.36	0.088
C8-C9	π	1.64743	N2-C13	π*	0.08787	17.39	0.38	0.078
C8-C9	π	1.64743	C3-C11	π*	0.38048	29.3	0.34	0.09
C8-C9	π	1.64743	C4-C6	π*	0.28589	27.25	0.36	0.09
C21-C29	π	1.66789	C22-C24	π*	0.31793	27.82	0.36	0.09
C21-C29	π	1.66789	C26-C27	π*	0.34192	27.8	0.36	0.09
C22-C24	π	1.67315	C21-C29	π*	0.34688	28.91	0.36	0.092
C22-C24	π	1.67315	C26-C27	π*	0.34192	29.53	0.36	0.093
C26-C27	π	1.6574	C21-C29	π*	0.34688	29.39	0.36	0.092
C26-C27	π	1.6574	C22-C24	π*	0.31793	27.13	0.36	0.089
LP-Cl1		1.92939	C3-C11	π*	0.38048	15.67	0.42	0.079
C3-C11	π	1.61952	C4-C6	π*	0.28589	148.86	0.02	0.093
C3-C11	π	1.61952	C8-C9	π*	0.36153	245.4	0.02	0.1
C8-C9	π	1.63847	N2-C13	π*	0.08787	56.66	0.03	0.072

Table S9. NBO analysis for imine **III** at the M06-2X/6-311+G(d,p) level.

IMINE III								
Donor (i)	Type	ED/e	Acceptor	Type	ED/e	E(2) (kcal/mol)	E(j)-E(i) (a.u.)	F(i,j) (a.u.)
N2-C13	π	1.94768	C5-C12	π*	0.36191	9.16	0.45	0.062
C5-C12	π	1.64739	N2-C13	π*	0.08805	17.63	0.38	0.079
C5-C12	π	1.64739	C7-C9	π*	0.37935	29.22	0.34	0.089
C5-C12	π	1.64739	C10-C36	π*	0.28565	27.15	0.36	0.09
C7-C9	π	1.67954	C5-C12	π*	0.36191	27.21	0.38	0.091
C7-C9	π	1.67954	C10-C36	π*	0.28565	24.35	0.38	0.087
C10-C36	π	1.66702	C5-C12	π*	0.36191	26.89	0.36	0.088
C10-C36	π	1.66702	C7-C9	π*	0.37935	30.62	0.34	0.092
C15-C30	π	1.74014	C16-C25	π*	0.44823	21	0.36	0.082
C15-C30	π	1.74014	C26-C28	π*	0.23794	20.65	0.41	0.083
C16-C25	π	1.55929	C15-C30	π*	0.24539	20.7	0.37	0.083
C16-C25	π	1.55929	C17-C19	π*	0.24863	22.16	0.35	0.082
C16-C25	π	1.55929	C21-C23	π*	0.24185	21.55	0.35	0.082
C16-C25	π	1.55929	C26-C28	π*	0.23794	18.97	0.39	0.081
C17-C19	π	1.75729	C16-C25	π*	0.44823	20.91	0.37	0.083
C17-C19	π	1.75729	C21-C23	π*	0.24185	22.57	0.38	0.083
C21-C23	π	1.75135	C16-C25	π*	0.44823	21.39	0.37	0.083
C21-C23	π	1.75135	C17-C19	π*	0.24863	24.02	0.38	0.085
C26-C28	π	1.75765	C15-C30	π*	0.24539	21.66	0.4	0.083
C26-C28	π	1.75765	C16-C25	π*	0.44823	21.75	0.37	0.084
LP-Cl1	€	1.92892	C7-C9	π*	0.37935	15.73	0.42	0.079
C7-C9	π	1.62065	C5-C12	π*	0.36191	252.75	0.02	0.1
C7-C9	π	1.62065	C10-C36	π*	0.28565	149.02	0.02	0.093
C15-C30	π	1.75461	C16-C25	π*	0.44823	245.9	0.01	0.104
C16-C25	π	1.55177	C15-C30	π*	0.24539	101.23	0.04	0.091
C16-C25	π	1.55177	C21-C23	π*	0.24185	292.8	0.01	0.091
C16-C25	π	1.55177	C26-C28	π*	0.23794	72.57	0.05	0.091

Table S10. NBO analysis for imine **IV** at the M06-2X/6-311+G(d,p) level.

IMINE IV								
Donor (i)	Type	ED/e	Acceptor	Type	ED/e	E(2) (kcal/mol)	E(j)-E(i) (a.u.)	F(i,j) (a.u.)
N1-C24	π	1.9462	C26-C27	π*	0.36304	9.26	0.44	0.062
C2-C5	π	1.67558	C7-C9	π*	0.32368	28.98	0.36	0.091
C2-C5	π	1.67558	C11-C20	π*	0.34857	27.44	0.37	0.09
C7-C9	π	1.68137	C2-C5	π*	0.32006	27.31	0.36	0.089
C7-C9	π	1.68137	C11-C20	π*	0.34857	28.26	0.37	0.092
C11-C20	π	1.65203	C2-C5	π*	0.32006	28.88	0.36	0.091
C11-C20	π	1.65203	C7-C9	π*	0.32368	27.95	0.35	0.089
C26-C27	π	1.64785	N1-C24	π*	0.09204	17.56	0.38	0.078
C26-C27	π	1.64785	C29-C31	π*	0.38051	29.2	0.34	0.089
C26-C27	π	1.64785	C32-C34	π*	0.2847	27.16	0.36	0.09
C29-C31	π	1.68024	C26-C27	π*	0.36304	27.16	0.38	0.091
C29-C31	π	1.68024	C32-C34	π*	0.2847	24.28	0.38	0.087
C32-C34	π	1.66541	C26-C27	π*	0.36304	26.9	0.36	0.088
C32-C34	π	1.66541	C29-C31	π*	0.38051	30.8	0.34	0.092
LP-N1	€	1.91259	C24-H25	σ*	0.04306	14.29	0.89	0.101
LP-Cl1	€	1.92911	C29-C31	π*	0.38051	15.71	0.42	0.079
C7-C9	π	1.67632	C11-C20	π*	0.34857	346.64	0.01	0.097
C26-C27	π	1.63696	N1-C24	π*	0.09204	60.62	0.02	0.072
C29-C31	π	1.61949	C26-C27	π*	0.36304	254.1	0.02	0.1
C29-C31	π	1.61949	C32-C34	π*	0.2847	145.99	0.02	0.093

Table S11. NBO analysis for imine **V** at the M06-2X/6-311+G(d,p) level.

IMINE V								
Donor (i)	Type	ED/e	Acceptor	Type	ED/e	E(2) (kcal/mol)	E(j)-E(i) (a.u.)	F(i,j) (a.u.)
N2-C14	π	1.94728	C4-C7	π*	0.36172	9.25	0.44	0.062
C4-C7	π	1.64741	N2-C14	π*	0.08789	17.42	0.38	0.078
C4-C7	π	1.64741	C5-C9	π*	0.28584	27.24	0.36	0.09
C4-C7	π	1.64741	C11-C12	π*	0.38062	29.31	0.34	0.09
C5-C9	π	1.66645	C4-C7	π*	0.36172	26.96	0.36	0.088
C5-C9	π	1.66645	C11-C12	π*	0.38062	30.72	0.34	0.092
C11-C12	π	1.68058	C4-C7	π*	0.36172	27.06	0.38	0.091
C11-C12	π	1.68058	C5-C9	π*	0.28584	24.39	0.38	0.087
C16-C22	π	1.69451	C19-C26	π*	0.37515	23.99	0.35	0.083
C16-C22	π	1.69451	C20-C24	π*	0.30062	28.51	0.37	0.091
C19-C26	π	1.67858	C16-C22	π*	0.33811	29.81	0.38	0.095
C19-C26	π	1.67858	C20-C24	π*	0.30062	22.65	0.38	0.083
C20-C24	π	1.70751	C16-C22	π*	0.33811	25.35	0.37	0.087
C20-C24	π	1.70751	C19-C26	π*	0.37515	29.66	0.35	0.093
LP-Cl1		1.92934	C11-C12	π*	0.38062	15.69	0.42	0.079
LP-N2		1.9114	C14-H15	σ*	0.04297	14.12	0.88	0.1
LP-O3		1.85681	C19-C26	π*	0.37515	35.2	0.45	0.119
C4-C7	π	1.63828	N2-C14	π*	0.08789	56.34	0.03	0.072
C11-C12	π	1.61938	C4-C7	π*	0.36172	245.58	0.02	0.1
C11-C12	π	1.61938	C5-C9	π*	0.28584	148.58	0.02	0.093
C19-C26	π	1.6249	C16-C22	π*	0.33811	223.53	0.02	0.093
C19-C26	π	1.6249	C20-C24	π*	0.30062	244.68	0.02	0.095

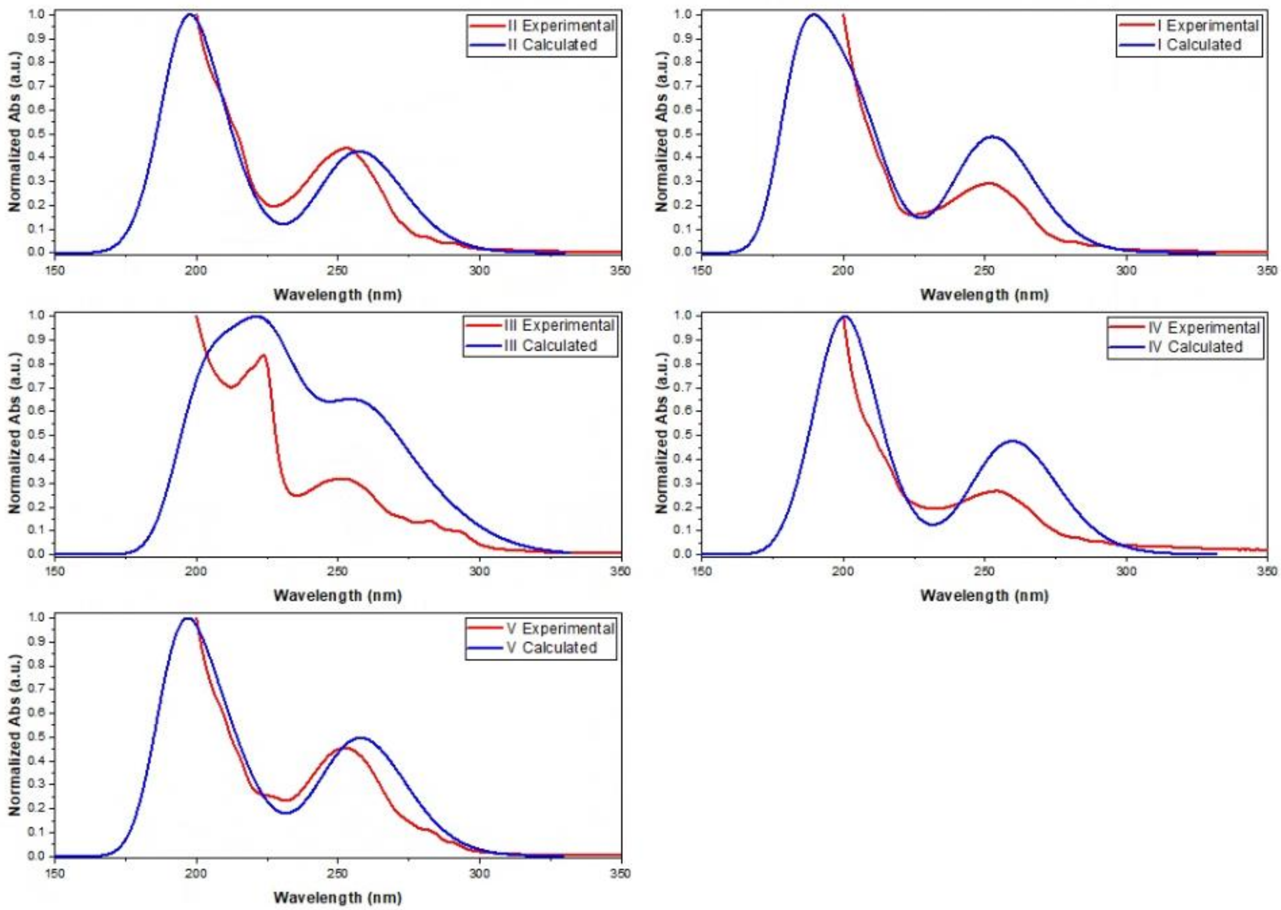

Figure S33. Comparison of experimental (red) and M06-2X/6-311+G(d,p) calculated (blue) UV-Vis spectra for the chiral imines **I-V**.
